# Ift46 deficiency causes renal cyst via enhanced Limk2 through lack of autophagy flux

**DOI:** 10.1186/s12964-026-02715-4

**Published:** 2026-02-12

**Authors:** Jae Hee Jun, Je Yeong Ko, Eun Ji Lee, Jinui Min, Hyeri Choi, Gyu Ran Lee, Sungju Jung, Kyung Hyun Yoo, Jong Hoon Park

**Affiliations:** 1 Molecular Medicine Laboratory, Department of Biological Sciences, Sookmyung Women’s University, Seoul, 04310 Republic of Korea; 2 Laboratory of Biomedical Genomics, Department of Biological Sciences, Sookmyung Women’s University, Seoul, 04310 Republic of Korea; 3 Research Institute of Women’s Health, Sookmyung Women’s University, Seoul, 04310 Republic of Korea

**Keywords:** Ift46, Limk2, PKD, EMT, Autophagy

## Abstract

**Background:**

Intraflagellar transport protein 46 (IFT46) is a core protein of the IFT B complex linked to signal transduction systems with primary cilia. Although the function of Ift46 has been extensively fully investigated, the study of Ift46 other than ciliogenesis has not. Recent studies have shown that Ift46 could control autophagy progression in addition to the mechanism of cilia formation.

**Methods:**

To investigate the biological function of Ift46, we performed and analyzed total RNA sequencing using Ift46 knockdown mouse inner medullary collecting duct cells. Gene ontology analysis revealed that Ift46 is associated with partial epithelial–mesenchymal transition (EMT). In vitro experiments demonstrated that Ift46 regulated partial EMT, LIM domain kinase 2 (Limk2) and autophagy flux. The direct interaction between Limk2 and p62/sequestosome 1 was verified through co-immunoprecipitation, and various drugs affecting autophagy were found to impact Limk2 stability. The role of Ift46, autophagy, and Limk2 in the renal cyst formation and the progression of EMT was highlighted using an in vitro three-dimensional culture system. Furthermore, the Ift46-autophagy-Limk2 axis was validated in a collecting duct-specific Ift46-deficient mouse and in human autosomal dominant polycystic kidney disease.

**Results:**

Ift46 regulated autophagy in mouse collecting duct cells, and Ift46 deficiency surprisingly led to an increase in Limk2 translation. Moreover, the translational changes in Limk2 were influenced by the regulation of autophagy. In Ift46-deficient mice, which exhibit a polycystic kidney disease phenotype, the upregulated Limk2 promoted mesenchymal characteristics despite minimal epithelial changes. Furthermore, the induction of autophagy suppressed Limk2, leading to the proposal of the ‘Ift46-autophagy-Limk2’ axis.

**Conclusions:**

Our study provides a novel finding that Ift46-regulated autophagy controls partial EMT through Limk2 regulation, suggesting a Limk2-dependent mechanism of cystogenesis in PKD.

**Supplementary Information:**

The online version contains supplementary material available at 10.1186/s12964-026-02715-4.

## Background

Intraflagellar transport (IFT) protein moves along the axoneme in the basal body of the cilia and transmits external signals through the primary cilia [1]. There are IFT-B complexes for anterograde transport from the basal body to the ciliary tip, and the IFT-A complex for retrograde transport from the ciliary tip to the basis [2, 3]. Among these subunits, Ift46 plays a very important role in cilia formation and function as a member of IFT-B complex [4, 5]. Ift46 is also often recruited through direct interaction with IFT52 [6–8], and plays a significant role [9]. When there is a defect in Ift46, the IFT-B complex becomes unstable and is degraded [8], causing problems in the primary cilia located in the kidney and spinal canal [10]. Previous studies have demonstrated that Ift46 defects in renal epithelial cells can induce cystogenesis without the aid of other cystogenesis-inducing factors [10]. In PKD, renal function is weakened by increased cell proliferation and subsequent cyst formation, and fibrosis of surrounding cells progresses [11–14]. 

LIM domain kinase (Limk) 2 belongs to the LIM kinase family, which includes Limk-1 and Limk-2 [15] and Limk2 is primarily regulated by Rho-GTPasee [16]. When Limk2 is phosphorylated by Rho-GTPase, it regulates the actin cytoskeleton by phosphorylating Cofilin, which controls actin filaments dynamics [17]. Most studies have shown that Limk2 conducts an oncogenic function in cancer and is particularly involved in promoting EMT [18–21]. 

In this study, we proposed a new mechanism by which Ift46 controls the PKD phenotype by regulating Limk2 through autophagy. Ift46 regulated autophagy in a ciliary-independent manner. Additionally, decreased Ift46 suppressed autophagy flux, leading to an increase in Limk2 and p62 expression. The promotion of EMT caused by enhanced Limk2 further aggravated cystogenesis. Therefore, we propose a new approach to understand the renal cyst formation mechanism in depth.

## Methods

### Cell culture

Mouse inner medullary collecting duct (mIMCD) were cultured in Dulbecco’s modified Eagle’s medium (DMEM)/F12 medium (LM 002–08, Welgene, Korea) and HEK293T cells were cultured in DMEM (LM001-05, Welgene). Both cells’ medium supplemented with 1% penicillin/streptomycin (LM002-04, Welgene) and 10% fetal bovine serum (FBS) (16000-044, Gibco). Culture conditions were maintained at 5% CO2, 37 °C, and humidity using an incubator. Cells were transfected with small interference RNA (siRNA) for identifying loss-of-function using Lipofectamine RNAiMAX Transfection Reagent (Invitrogen™, 13778150) and for identifying gain-of-function using FuGENE^®^ HD Transfection Reagent (Promega, E2311) with a constructed vector. siRNA and drugs used in this study were described in Supplementary Table 1.

### Western blotting and qRT-PCR

RNA and proteins were isolated from cell lysates and kidney tissues using Nucleo Spin RNA/Protein kits (740933.250, Macherey-Nagel). For electrophoresis, 30–70 µg of protein was loaded into a sodium dodecyl sulfate-polyacrylamide gel electrophoresis (SDS-PAGE) gel with a protein marker (PM2610, SMOBiO). Primary antibodies were incubated overnight in 1% skim milk (232100, BD Difco). The antibodies used in this study were described in Supplementary Table 1. The next day, secondary antibodies were incubated for over 1 h and chemiluminescent signals were detected using AmershamTM ImageQuantTM 800 with EzWestLumi plus reagent (2332637, Atto). The concentration of RNA extracted from cell lysates and tissues was measured using the NanoDropOne spectrophotometer (Thermo Fisher Scientific), and 2–5 µg of RNA was reverse transcribed. Using SYBR green, amplified cDNA by LightCycler 96 system (Roche) was quantified.

### Cell counting Kit-8 (CCK-8) assayC

Transfected cells were reseeded in a 96-well plate, and cells were incubated with complete medium. Then, the medium was replaced with a mixed solution with CCK-8 (Ck04-05, Dojindo) and DMEM/F12 at 1:10 ratio in a 12-h cycle or daily cycle and incubated for 1–2 h. After transferring to a new 96-well plate, absorbance was measured using and enzyme-linked immunosorbent assay plate reader.

### Tubule formation assay

mIMCDs (3.0 × 10^5) were suspended in 250 µL of a mixed solution of 10x DMEM (12800-017, Gibco) and 10x reconstitution buffer. Suspended cells were mixed with 1 mL of collagen (354236, BD) and then quickly neutralized using 2 µL of 10 N NaOH. The suspension was spread evenly on a 12-well plate, and after the gel hardened, complete media was added, and it was observed under a microscope 3–5 days later.

### Immunocytochemistry

mIMCDs were grown on a cover glass and fixed with methanol at − 20 °C. The primary antibody was incubated overnight at 4 °C with a permeabilizing solution and a secondary fluorescent antibody with non-overlapping wavelengths was incubated the next day. After fixing to the slide glass with a mounting solution. For analyze autophagy flux, cells were transfected using Premo Autophagy Tandem Sensor RFP-GFP-LC3B Kit (P36239, Invitrogen™) for 24 h and fixed on slide glass after labeling with DAPI. To observe the autophagy flux in live cells, we used the CYTO-ID^®^ Autophagy detection kit (ENZ-51031-K200, ENZO). Cells were observed using a confocal fluorescence microscope (LSM-700, Carl-Ziess at the Chronic and Metabolic Diseases Research Center, Sookmyung Women’s University) and analyzed using ImageJ and Zen software (Carl Zeiss).

### Co- Immunoprecipitation

We prepared cells transfected with each vector for 48 h and cells were washed with 1×PBS and then incubated with lysis buffer at 4 °C for 5 min. Lysates were collected through centrifugation at 4 °C for 15 min and incubated with antibodies using a rotator at 4 °C overnight. The antibodies were described in Supplementary Table 1. On the next day, Dynabeads™ Protein G (10004D, Invitrogen) or Dynabeads™ Protein A (10001D, Invitrogen) was added at room temperature for 2 h, and the precipitates were washed three times. Following electrophoresis, precipitates were loaded into 8% SDS-PAGE gels.

### Mouse model

Mouse kidney tissue samples were obtained from crossing IFT46-floxed with HoxB7-cre at postnatal day 18. For immunofluorescence, paraffin-embedded mouse kidneys were cut into thin slices on slide glasses, and primary and fluorescent secondary antibodies were incubated and observed through confocal fluorescence microscope. In vivo experimental procedures were conducted under review and approval of the IACUC at Sookmyung Women’s University, Seoul, Republic of Korea, and the number of an acceptance is SMWU-IACUC-2011-011-02.

### Human autosomal dominant polycystic kidney disease (ADPKD) kidney

To support our hypothesis, we used human kidney paraffin-embedded tissue blocks obtained from Seoul National University Hospital and Samsung Medical Center. All experiments were managed in accordance with the Declaration of Helsinki, and each approval number is the Institutional Review Board of Seoul National University Hospital (IRB number: H-0701–033–195) and Samsung Medical Center (IRB number: SMC 2019–08–074). Kidney samples were surgically resected after approval, and in particular, noncancerous tissue regions were obtained from patients with renal cell carcinoma who exhibited radical nephrectomy as a control group.

### Total RNA sequencing

Total RNA sequencing was performed by Geniens Korea using mRNA produced in our laboratory. Total mRNA was extracted from mIMCD transfected with control siRNA or Ift46 siRNA during 48 h. Workflow of total RNA sequencing is described as a pipeline in Supplementary Fig. 1A.

### In vitro three-dimensional 3D culture and immunocytochemistry

The system consisted of reseeding transfected mIMCD on 8-well chambered slides with a 1:1 ratio of matrigel (354230, Corning) and medium containing cells. Serum free medium was replaced with forskolin daily for 5–6 days, images of forming cysts were taken with an optical microscope. Spheroids were fixed for 30 min with 10% paraformaldehyde, washed five times with Dulbecco’s PBS (DPBS), and incubated with permeabilization buffer for 30 min at room temperature. Spheroids were mounted and were observed using a confocal fluorescence microscope.

### Statistics

All statistical analyses were presented as the mean ± standard deviation (SD). For 3D culture data (cystogenesis and tubulogenesis), data were presented as the median with interquartile range. t-test was performed using GraphPad Prism 5.0 (GraphPad software). Studies using cells were repeated at least three times. P-values less than 0.05 were considered statistically significant. *P < 0.05, **P < 0.01, ***P < 0.001, ****P < 0.0001. n.s. means no-significant.

## Results

### Decreased Ift46 promotes partial epithelial-to-mesenchymal transition in normal kidney epithelial cells

**Fig. 1:**
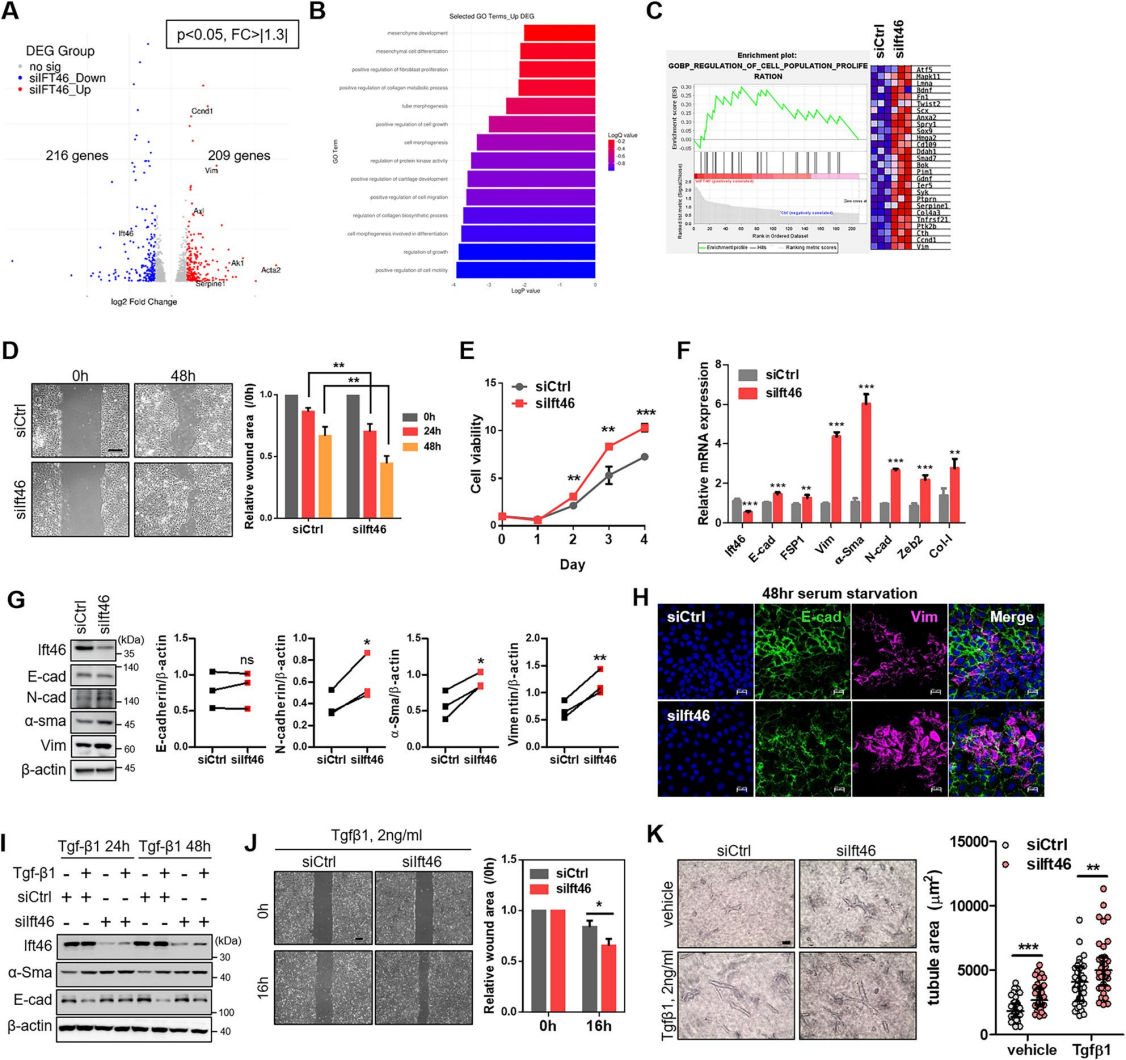
Reduced Ift46 caused partial epithelial-to-mesenchymal transition.**A** Volcano plot graph produced by analyzing total RNA sequencing with Ift46 knockdown mIMCD. Red dots were upregulated DEGs, and blue dots were downregulated DEGs. Names of our genes of interest were marked in the graph. **B** Upregulated GO term list produced by analyzing total RNA sequencing with Ift46 knock-down mIMCD. Length of the bar indicated log-p-value, and color variation indicated log Q value. **C** GSEA analysis showed upregulation of cell population and proliferation under Ift46 silencing. **D** The representative images show migrated cells using wound-healing assay under Ift46 knockdown. Quantification value of the migration degree is presented in the graph on the right. Scale bar, 100 μm. **E** The potential of viable cells was counted using cell-counting kit (CCK-8) in Ift46 silencing mIMCD. Measurements were taken once a day at the same time. **F** Relative mRNA expression of EMT markers in reduced Ift46 is shown graphically. Epithelial-cadherin (E-cad) marked epithelial cells, and S100 calcium binding protein A4 (FSP1), neural cadherin (N-cad), alpha-smooth muscle actin (α-Sma), zinc finger E-box binding homeobox 2 (Zeb2), collagen, type I, alpha 1 (Col-I) and vimentin (Vim) marked mesenchymal cells. **G** Epithelial-to-mesenchymal transition marker change with Ift46 silenced mIMCD cells using western blotting analysis. Graph shows relative band intensities of E-cadherin, N-cadherin, α-Sma, and Vimentin. Graph analysis performed using paired t-test. **H** Fluorescent staining images to identify EMT markers in Ift46 knocked-down. mIMCD cells were subjected to Ift46 silencing and serum starvation at 48 h. **I** Western blotting analysis shows epithelial-to-mesenchymal transition in Ift46 knockdown with 24or 48 h of treatment with TGF-β1. **J** Cell migration degrees were measured using wound-healing assay in Ift46 silencing with 20 ng/ml TGF-β1 for 16 h. The graph on the right displays the quantification of images obtained using wound-healing assay. Scale bar, 100 μm. **K** Tubular morphogenesis of Ift46 silenced mIMCD cells with or without TGF-β1 using an in vitro 3D culture system. Scale bar, 100 μm. The graph was quantified measuring tubule areas using ImageJ program, and representative images were selected by referring to the graph

We performed differential gene expression analysis of bulk RNA-sequencing data comparing Ift46 silencing samples with normal samples (using DESeq2 v1.40.2). Then, we obtained 216 results of downregulated differentially expressed genes (DEGs) and 209 results of upregulated DEGs (Supplementary Fig. 1A). Herein, DEGs were indicated by a volcano plot graph (Fig. 1A), and a heatmap (Supplementary Fig. 1B). Actin alpha 2 and vimentin, known as representative markers of mesenchymal transition, and Ccnd1, Glipr2, Ak1 and Serpine1, which show high correlation with EMT, increased in their fold-change value with Ift46 deficiency and especially displayed interesting genes in a volcano plot graph (Fig. 1A). Following Gene Ontology term and gene set enrichment analysis proposed the possibility that Ift46 deficiency was related to EMT progression (Fig. 1B, C). Down-regulated GO term analysis related to kidney development and epithelium morphogenesis (Supplementary Fig. 1C). In accordance with the results, we experimented on the relationship between Ift46 and EMT. Under decreased Ift46 condition, proportions of cell viability and restoring wound-healing were significantly increased (Fig. 1D and E). Next, we validated EMT-related gene expression, E-cadherin, as an epithelial marker, was not affected by Ift46, but the mesenchymal markers were significantly increased in the Ift46 defect (Fig. 1F, G). Immunofluorescence images also indicated potent vimentin signals in Ift46 silencing with serum starvation for 48 h (Fig. 1H) [22]. 

When TGF-β1 was treated [23, 24], although mesenchymal transition increased in Ift46 knockdown, no change in epithelial markers was confirmed and not only the resilience of the scratched cell matrix but also quantification of tubular epithelial cell area progressed faster under silenced Ift46 (Fig. 1I, J, K). These findings suggest that Ift46 absence not only initiates the partial EMT process but also accelerates EMT induced by TGF-b1 with a synergistic effect.

### Lack of Ift46 regulates partial EMT via Limk2 alteration

**Fig. 2:**
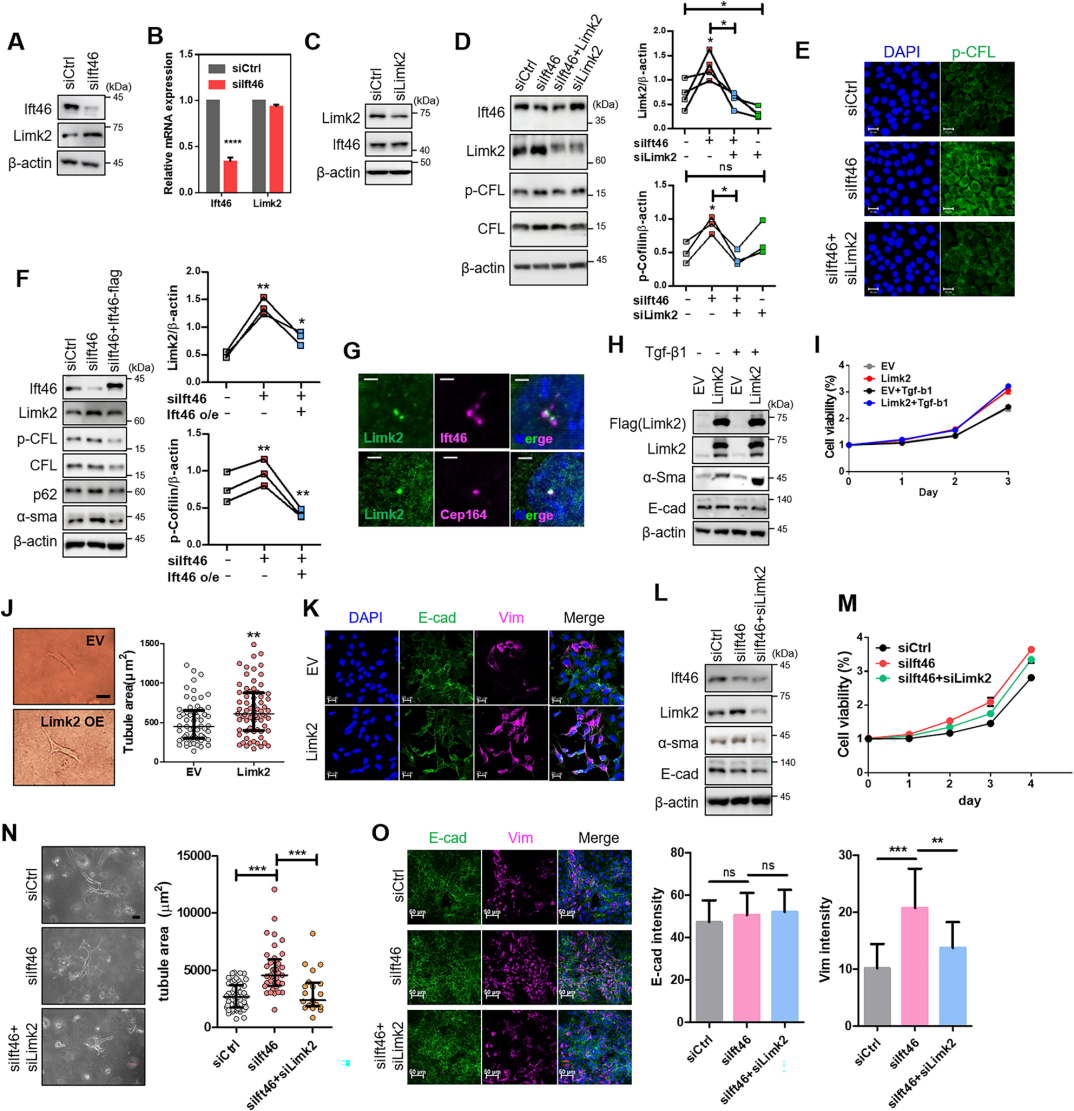
Ift46 modulates partial epithelial-to-mesenchymal transition via Limk2 alteration. **A** Expression of Limk2 under Ift46 knockdown using western blotting and (**B**) qRT-PCR analysis in mIMCD normal kidney collecting duct cells. **C** Western blot data confirmed translational expression of Ift46 due to Limk2 reduction. **D** Immunoblotting data showed Limk2 and its target, cofilin phosphorylation, expression altered by Ift46 and Limk2 existence. **E** Fluorescence images showed changes of phosphorylated cofilin upon Ift46 and Limk2 deficiency. **F** Restoration of Limk2, p-Cfl, p62, and α-Sma was observed using western blot analysis of Ift46 construct transfection with Ift46 silenced IMCD. **D**, **F** Graphs indicate relative band intensities of Limk2 and p-CFL. Graph analysis performed using paired t-test. **G** Immunofluorescence staining images show that Limk2 coexists with a portion of Ift46 and fully co-expressed with centrosome protein 164 (Cep164), as a centrosome marker in ciliated mIMCD cells. Scale bar, 2 μm. **H** Blotting analysis shows EMT change under overexpressed Limk2 with or without TGF-β1, EMT marker; E-cadherin, epithelial marker; α-Sma (alpha-smooth actin), mesenchymal marker. **I** The potential of viable cells was counted using cell-counting kit (CCK-8) in overexpressed Limk2 with or without TGF-β1 during days. **J** Tubular morphogenesis of overexpressed Limk2 in mIMCD using an in vitro 3D culture system. Scale bar, 100 μm. Each dot in the bar graph represents the tubule area, which was quantified by measuring the area of branching mIMCD morphology in collagen gel. **K** EMT marker confirmation in overexpressed Limk2 using immunofluorescence staining experiments. **L**–**O** Phenotypes of epithelial-to-mesenchymal transition by Ift46 silencing and double knockdown of Ift46 and Limk2 in mIMCD cells. **L** Immunoblot analysis shows epithelial marker changes with Limk2 silencing and double knockdown of Ift46 and Limk2. **M** Cellular viability was measured using CCK-8 assay for four days under Ift46 knockdown and double knockdown of Ift46 and Limk2. **N** Alteration of tube morphogenesis under Ift46 and Limk2 expression changes, and statistics for the data shown in tube morphogenesis images are on the right. Scale bar, 100 μm. **O** Immunofluorescence staining images show restoration of vimentin intensity by double knockdown of Ift46 and Limk2. DAPI (blue), E-cadherin (green), and vimentin (magenta). The graph shows the intensity of E-cadherin and Vimentin from three independent experiments

While further exploring another function of Ift46 other than ciliogenesis, we made a new discovery related with Limk2 [25]. To specifically investigate the non-ciliary function of IFT46, experiments were conducted under conditions maintaining cells in a non-ciliated state using complete medium. Translational expression of Limk2 in Ift46-deficient mIMCD was increased, but transcriptional expression did not change and it was unidirectionally in superior phase (Fig. 2A-C). However, Ift46 did not directly interact with Limk2 (Supplementary Fig. 2). We next confirmed phosphorylated cofilin (p-Cfl) increasing by Ift46 knocked-down [25], but p-Cfl was not increased when Ift46 and Limk2 were simultaneously silenced (Fig. 2D, E). Following that, overexpression of Ift46 in the Ift46 silencing state restored Limk2 and p-Cfl to their original expression levels (Fig. 2F). Limk2 partially coexisted with Ift46 in primary cilia-induced cells, and coincided with Cep164, a centrosome marker (Fig. 2G). Thus, these results suggest that Ift46 is an unmistakable target for regulating the activity of Limk2.

Since it has been reported that cofilin mediates EMT [26], we readily hypothesized the relationship between Limk2 and partial EMT. Limk2 overexpression lead to increased α-Sma, but E-cad did not change, and the more synergistic effect of EMT due to the addition of TGF-β1 showed similar EMT inclination (Fig. 2H). Moreover, the outcome of Limk2 overexpression was identical with the silencing of Ift46, as shown in Fig. 1I, which enhanced cell viability, expanded tubule areas, and strengthened the intensity of vimentin (Fig. 2I, J, K, Supplementary Fig. 3), hence Limk2 promoted partial EMT as reduced Ift46 did. Conversely, Limk2 deficiency declined even TGF-β1-induced EMT (Supplementary Fig. 4).

As shown by Limk2 expression in Fig. 2D, α-Sma expression, cell viability and tubule morphogenesis declined compared with Ift46 single knockdown, and fluorescence intensity of vimentin also restored (Fig. 2L-O). Altogether, these findings suggest that reduced Ift46 promotes EMT by mediating enhanced protein expression of Limk2.

### Ift46 controls autophagy, which is inversely associated with Limk2

**Fig. 3:**
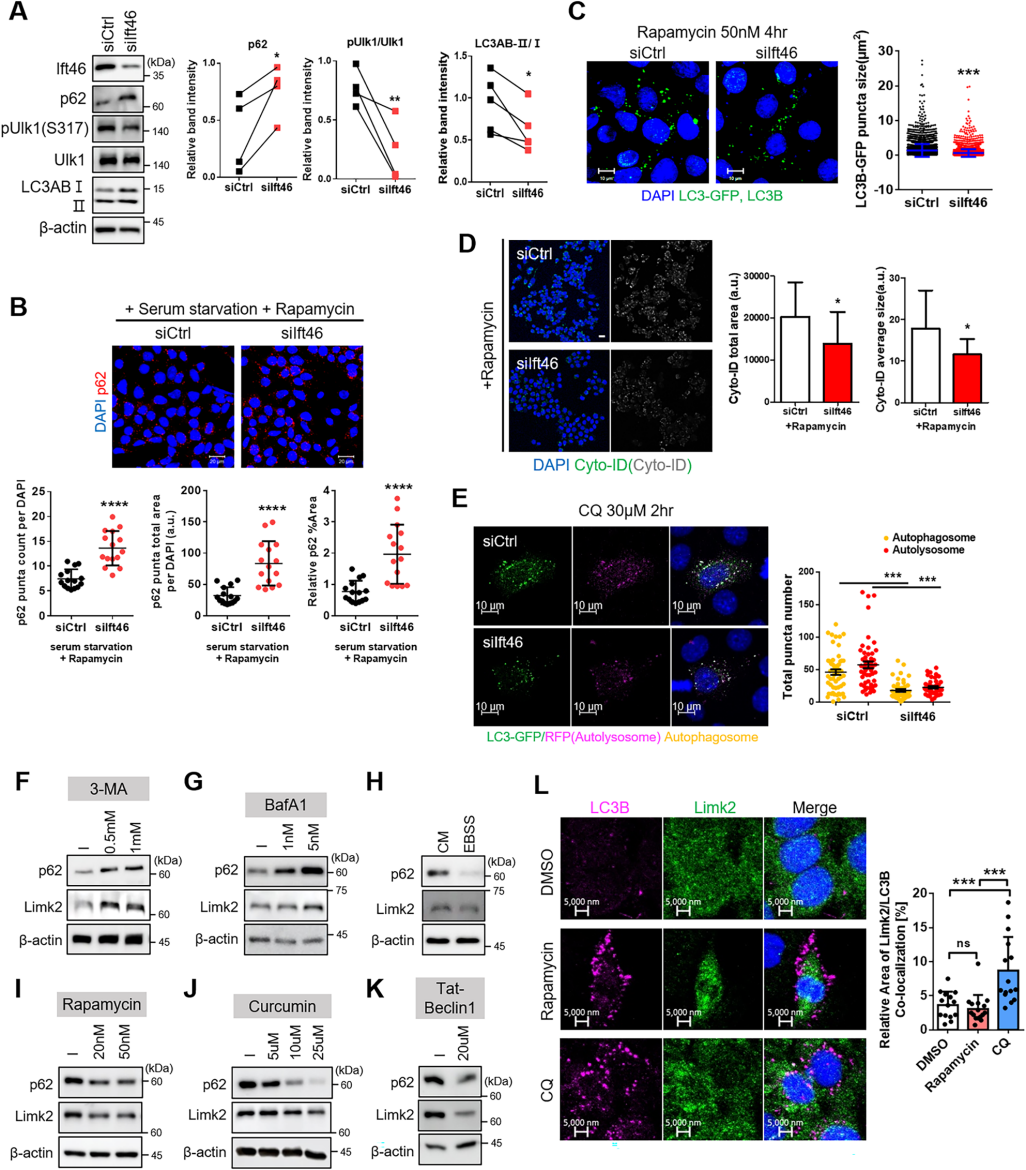
Suppressed autophagy flux by Ift46 blockage and fluctuated Limk2 via treatment with autophagy inducers and inhibitors. **A** Switch of autophagy in Ift46 knocked down analyzed using immunoblotting, and graphs indicate relative band intensities of p62, pUlk1 to Ulk1 and LC3ABⅡ to Ⅰ. Graph analysis performed using paired t-test. **B** Confirmation of p62 puncta upon Ift46 existence under serum starvation and rapamycin-induced autophagy. After Ift46 knock-down, brief serum starvation and 50 nM rapamycin treatment for 4 h. Images represent puncta of p62, and graphs below demonstrate the number, total area of puncta, and relative percentage of area through p62 puncta measured using ImageJ tool. Scale bars in the right bottom were 20 μm. **C** Representative immunofluorescence images show LC3B puncta for autophagy flux through GFP-tagged LC3 vector transfection and LC3B immunostaining in Ift46 knockdown with induced autophagy by rapamycin treatment. LC3B-GFP puncta. The graph on the right represents quantification of LC3B-GFP puncta size in Ift46 control and knockdown using ImageJ tool. **D** The autophagy flux change relying on Ift46 was verified using CYTO-ID detection experiment. Confocal images show a decrease of CYTO-ID due to lack of Ift46, and it implies autophagic vacuoles reduction. CYTO-ID was stained green, and images converted to monotone for measurement were attached side by side. Right graphs show quantification of CYTO-ID vacuoles total area and average size using imageJ. Scale bar, 20 μm. **E** Autophagy tandem sensor RFP-GFP-LC3B puncta indicate autophagy flux in Ift46 silencing with short exposure of CQ for holding autophagosomes. Immunofluorescence stained images stand for autophagy flux diminish under Ift46 blockage. General LC3 expressed green fluorescence-light, and acidic LC3 by lysosomal fusion turned out red fluorescence-light. Yellow LC3 puncta signified the autophagosomes, as fused green and red fluorescence-light. The graph on the right assessed the number of red puncta (autolysosome) and yellow puncta (autophagosome). Scale bars in the left bottom of immunofluorescence images were 10 μm. **F**–**G** Weakened autophagy by drug treatment increases Limk2 expression confirmed using western blot analysis. All types of drugs for autophagy blockage were incubated for 4 h with various concentration. **F** 3-MA (3-methyladenine) treatments enhanced expression of Limk2 during autophagy weakening. **G** BafA1 (Bafilomycin A1) attenuated autophagy process for gradient concentration. (**H**–**K**) Decreased Limk2 expression by induced autophagy, and autophagy activity was confirmed by p62, a representative autophagy marker. **H** Induced autophagy via culture in EBSS (Earle’s balanced salt solution)-starvation for short time, 2 h. Control group was cultured in complete medium with 10% FBS. **I** Immunoblotting analysis data show that rapamycin-induced autophagy weakened Limk2. Rapamycin treatment (20 and 50 nM) for 4 h. **J** Induced autophagy attenuated Limk2 expression depending on the concentration of curcumin treatment for 4 h using immunoblotting analysis. **K** Treatment with Beclin-1 peptide within 4 h promotes autophagy in IMCD cells, and blotting analysis presented reduction of p62 and Limk2. **L** Immunofluorescence images showed localization of Limk2 depending on autophagy progression. Rapamycin 50 nM and CQ 30 µM treatment for 4 h, respectively. LC3B was stained in magenta, Limk2 was stained in green, and intersection was shown in yellow. Scale bars in the left bottom of immunofluorescence images were 5 μm. Right graph presented relative area of Limk2 and LC3B co-localization by using Zeiss Zen blue program. a.u. means arbitrary unit

In our previous study, the relationship between Ift46 and autophagy was observed [10]. First of all, p62 increased, phosphorylated Ulk1 at serine 317 was reduced in contrast to total Ulk1, and LC3A/B-Ⅱ/Ⅰ decreased in Ift46 knocked down mIMCD (Fig. 3A). We also observed the restoration of p62 to its original expression through the additional Ift46 effect (Fig. 2F). Following experiments of immunofluorescence staining treated with autophagy drugs since it was hard to captured moment of repeated cycle of autophagy process in basal condition [27]. The results of p62 puncta in Ift46-deficient cells briefly exposed to rapamycin with serum withdrawal showed a significant increase in the number, total area, and percentage area of p62 puncta (Fig. 3B). Quantification of green fluorescent protein (GFP)-tagged LC3 fluorescent and LC3B puncta, and CYTO-ID puncta in the absence of Ift46 with rapamycin treatment decreased (Fig. 3C and D). Furthermore, the autophagy flux due to Ift46 defects was observed using the Tandem Sensor RFP-GFP-LC3B autophagy assay kit. Both the yellow autophagosome puncta, where green and red intersect, and the red autolysosome fused with autophagosome and lysosome were reduced because Ift46 decrease (Fig. 3E). Therefore, the results revealed that Ift46 controls the autophagy flux.

Next, to investigate relationship between autophagy signaling and Limk2, we screened various autophagy drugs. First of all, 3-Methyladenine and, bafilomycin A1, which are widely used to attenuate autophagy, and translational expression of Limk2 were increased (Fig. 3F, G). Then, after autophagy promotion using the autophagy inducers rapamycin, curcumin, EBSS medium, and Becline1 peptide, Limk2 expression decreased (Fig. 3H, I, J, K, Supplementary Fig. 5A). We also checked the localization of Limk2 with LC3B depending on autophagy state. Limk2 localized in both the nucleus and cytosol in normal conditions, however, it was observed that big and little dots of LC3B did not overlap at all with Limk2 in rapamycin treatment, while Limk2 mainly remained in the cytosol (Fig. 3L, Supplementary Fig. 5B). Conversely, LC3B and Limk2 were overlapped in CQ treatment. Recent studies have suggested that the autophagy regulation was due to the interaction with IFT-B complexes [28–30], therefore we also verified each silencing effect of Ift20 and Ift88 promoted Limk2 and inhibited autophagy progression with or without serum (Supplementary Fig. 5C). In summary, expression of Limk2 is altered by the autophagy status and IFT-B proteins including Ift46, known to controlling autophagy, also affect Limk2.

### Sqstm1/p62, known as a classical autophagy receptor, directly interacts with Limk2

**Fig. 4:**
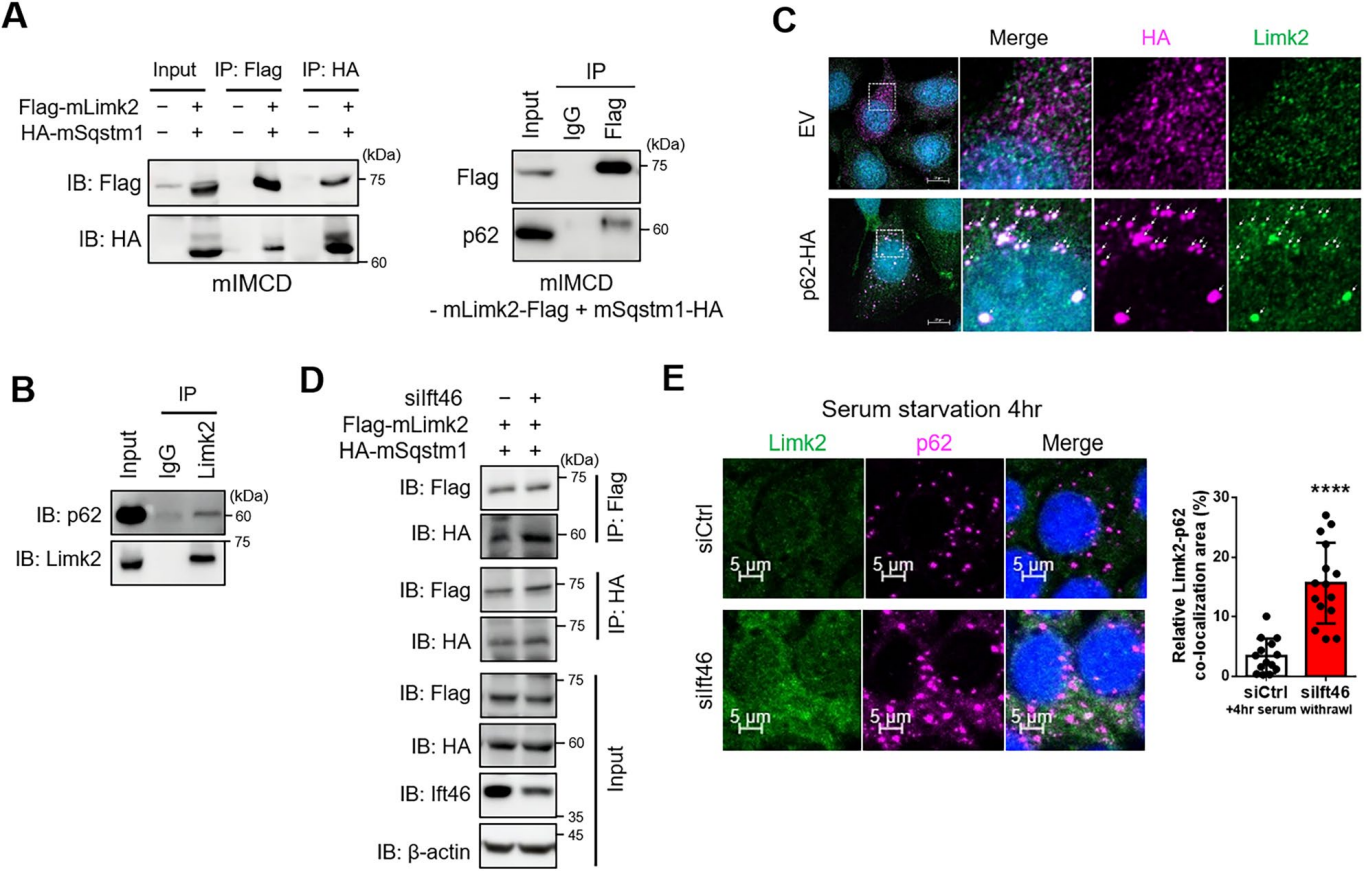
Limk2 directly interacts with the autophagy receptor p62/Sqstm1. **A**–**B** Immunoblotting data show alterations of p62 and Limk2 upon each expression changes. **A** Co-immunoprecipitation (Co-IP) assay is performed with flag-tagged Limk2 and HA-tagged p62/Sqstm1 transfected IMCD. The result reveals direct interaction between Limk2 and p62 under mouse renal collecting duct cell line. **B** Co-IP analysis result indicates that endogenous Limk2 interacts with p62 in mIMCD cell. **C** HA-tagged p62 shows high co-localization with Limk2. White arrows highlight punctate Limk2 signals that overlap with p62. The scale bars in the far-left panels represent 20 μm. **D** Co-IP analysis shows altered affinity between Limk2 and p62 upon Ift46 silencing in mIMCD cell. **E** Co-localization of Limk2 and p62 in the cytoplasm upon Ift46 silencing and serum starvation-induced autophagy. Scale bars in the left bottom of microscopy images were 5 μm. The graph on the right assessed relative Limk2 and p62 co-localization area percentage

As a consequence of the previous results, we investigated the relationship between p62 and Limk2. We identified the possibility that there is a high affinity between Limk2 and p62 [31], and then the interaction was investigated using co-IP assay. As expected, exogenous Limk2 and p62 were also strongly attracted in both mIMCD and HEK293T (Fig. 4A, Supplementary Fig. 6A). Endogenous Limk2 and p62 also directly interact (Fig. 4B). Furthermore, Limk2 co-localized with HA tagged p62, and even Limk2 comprised puncta-like forms (Fig. 4C). As Ift46 deficiency led to increased expression of p62 and Limk2, we subsequently investigated whether Ift46 modulates the interaction between p62 and Limk2. According to co-IP analysis, the interaction between Limk2 and p62 was further enhanced under Ift46 silencing (Fig. 4D). Limk2 also co-localized with p62 to a lesser extent during brief autophagy induction, but there was an increase cytosolic co-localization of Limk2 with p62 in Ift46 deficient cells (Fig. 4E). In addition, Limk2 and p62 increased under the deficient Ift46 condition with rapamycin, but not substantially as in the group without rapamycin (Supplementary Fig. 6B). Taken together, Limk2 directly binds to p62, and Ift46 depletion leads to persistent co-aggregation of p62 and Limk2.

### EMT and cyst formation are controlled by the ‘Ift46-autophagy-Limk2’ axis

**Fig. 5:**
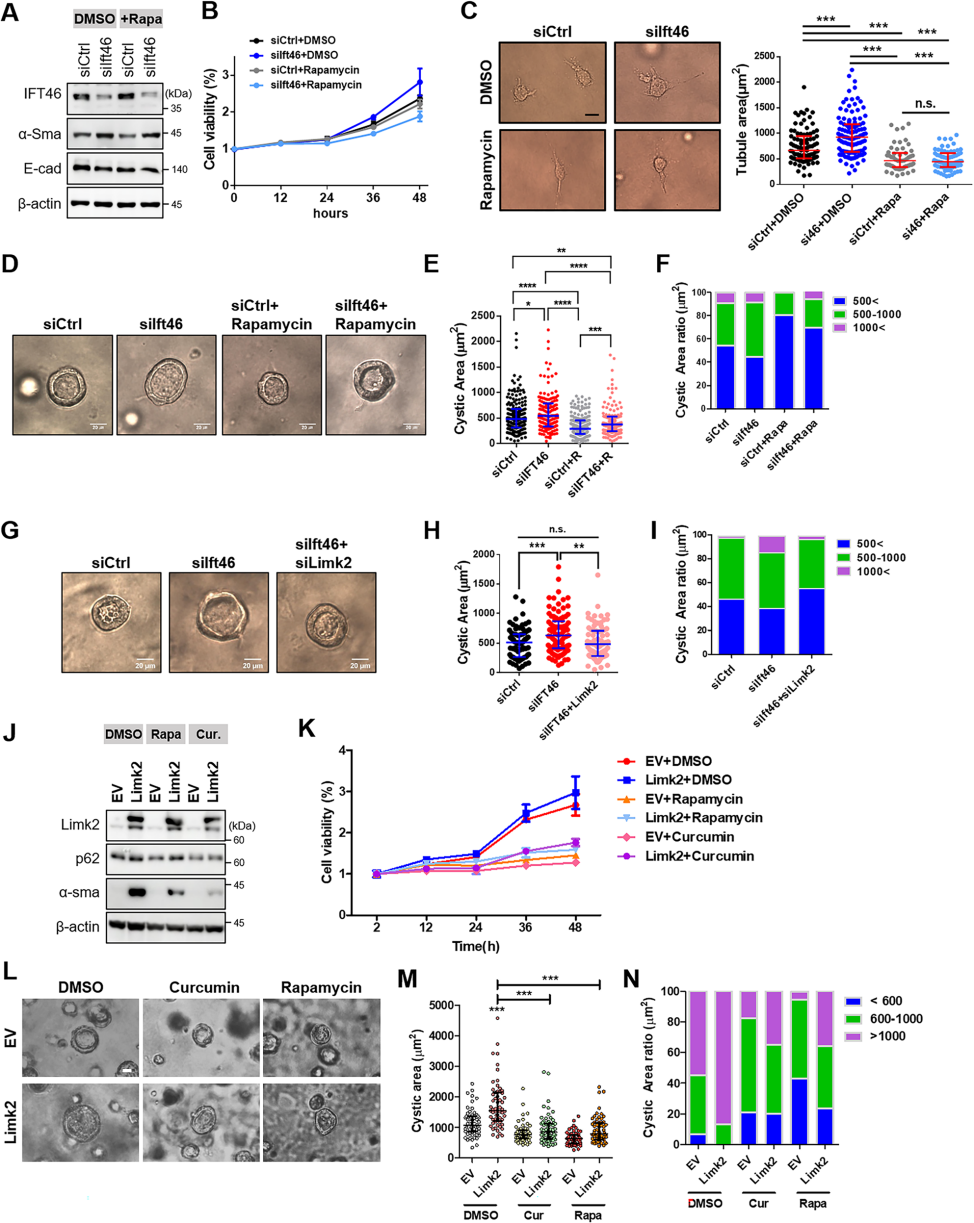
Axis of Ift46-autophagy-Limk2 controls EMT progression and cystogenesis. **A**–**C** Different types of analysis result in Ift46 silencing control EMT even upregulated the autophagy pathway. **A** Protein expression of α-Sma and E-cadherin changed by Ift46 knocked down with rapamycin-induced autophagy. **B** The graph indicates cellular viability under identical experiments using CCK-8 assay. **C** Induced autophagy by rapamycin treatment with Ift46 silencing changed tubular morphogenesis and quantification of tubule sizes. Scale bar, 20 μm. (**D-F**) The results show the ability to form cysts in in vitro 3D culture cysts under Ift46 knocked down for 48 h with induced autophagy by rapamycin treatment for 4 h in IMCD cells. The generated cyst size was measured on the 5th day of 3D culture. **D** The microscopic images represented cyst sizes in each condition. **E** Cystic lumens were measured using i-Solution software program. Approximately 50 or more images were taken and measured. **F** Bar graph shows the percentage of cyst size. The blue group was down to 500 µm^2^, the green group was 500 to 1000 µm^2^, and the red group was up to 1000 µm^2^. **G**–**I** The following results show cyst formation through an in vitro 3D system in Ift46 and Limk2 double knocked down IMCD. **G** Cysts were captured on day 5 after 3D culture under an optical microscope. The most representative image was selected through the statistics of the measured cyst. **H** Cystic areas are quantified and indicated by a dot graph. The graph shows estimated mean value of cystic area. **I** Cystic areas were classified based on three identical criteria as the previous experiment. Scale bars in the right bottom of microscopy images were 20 μm. **J** Western blots show change of α-Sma under over-expressed Limk2 with or without autophagy drug treatments. Concentration of rapamycin was 50nM, curcumin was 5µM and 4 h treatments. **K** Percentage of cell viability by CCK-8 assay under over-expressed Limk2 with or without treating autophagy drugs. CCK-8 assay was proceeded 2 h incubation for every 12 h. (**L-M)** Representative images showing cystic areas with Limk2 over-expressing and subjected autophagy drug treatment under equivalent conditions as in the previous experiment. ‘Rapa’ means rapamycin, ‘Cur.’ means curcumin. Scale bars in the right bottom of microscopy images were 20 μm. Graph shows difference of cystic area size . **N** The percentage of cystic area were calculated and classified three ranges: < 600 µm^2^, > 600 µm^2^ and < 1000 µm^2^, and > 1000 µm^2^

Since reduced Ift46 causes partial EMT (Figs. 1 and 2), we investigated whether the Ift46-autophagy-Limk2 axis controls EMT and renal cyst enlargement [32, 33]. In blotting analysis, cellular viability, and tubule morphogenesis, mesenchymal transition phenotypes also increased most significantly in Ift46 reduction and there was little difference in rapamycin treatment and Ift46 silencing (Fig. 5A, B, C). Although Ift46 silencing also resulted in enlarged cysts, but this effect was reduced under rapamycin treatment (Fig. 5D, E, F). Collectively, we showed alterations in EMT and cystogenesis under the control of Ift46 with autophagy induction. As was anticipated, the highest increase of viable cell percentage was shown in overexpressed Limk2 simultaneously with Ift46 silencing. Even autophagy was activated, Ift46 KD with Limk2 overexpression was higher, but there was no significant difference upon existences of Ift46 and Limk2 (Supplementary Fig. 7A). Similar results were also shown in the tube-morphogenesis, an increase in tubule size due to Ift46 KD was observed regardless of Limk2 in basal condition, and however, tubule size was slightly increased in Ift46 KD with over-expressing Limk2 and rapamycin rather than Ift46KD with rapamycin. (Supplementary Fig. 7B, C) Additionally, Ift46 and Limk2 dual knock-down repaired cyst expansion, and as a result, expression of Ift46 and Limk2 determined in vitro renal cyst phenotypes (Fig. 5G, H, I). The changes of α-Sma, p-Cfl and cystogenesis also confirmed by in vitro 3D culture experiments (Supplementary Fig. 7D, E). To discover the characteristics of EMT phenotype and cystogenesis through Limk2 with autophagy induction, and it was discovered that the highest increase of α-Sma expression, cellular viability and cyst formation was exhibited under Limk2 overexpression (Fig. 5J, K, L, M, N). Overexpressed Limk2 increased α-Sma expression, cellular viability, and tubule morphogenesis, but no significant increase was confirmed due to autophagy inducer treatment. Tat-Beclin1, a direct autophagy inducer, reduced cyst size upon treatment, indicating that proper induction of autophagy can suppress cyst growth (Supplementary Fig. 7F). In conclusion, modulating Limk2 and autophagy can attenuate partial EMT and cyst formation triggered by Ift46 deficiency.

### Expression of Ift46 and Limk2 in Ift46-defect mouse and patients with polycystic kidney disease

**Fig. 6:**
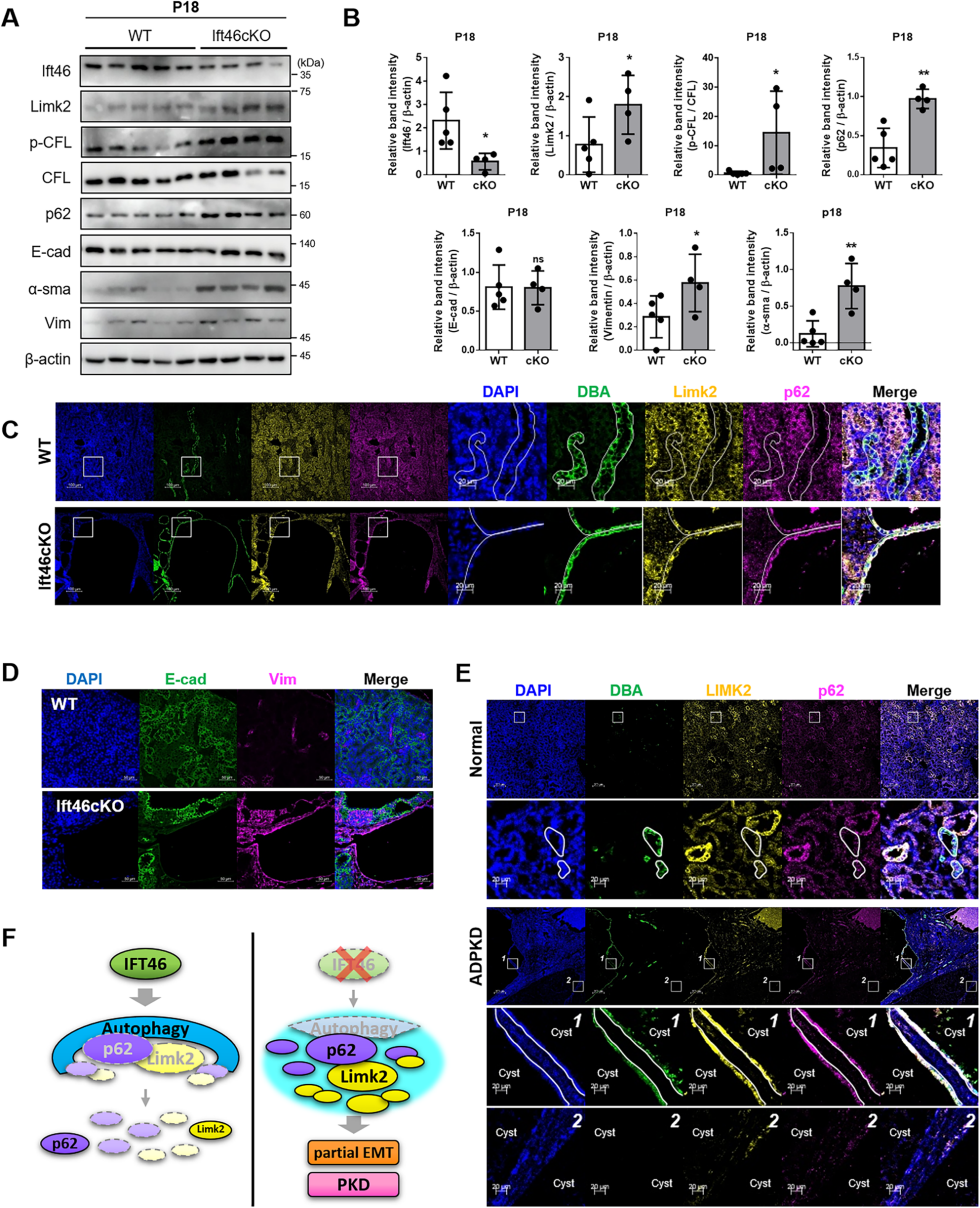
Increased Limk2 expression and epithelial-to-mesenchymal transition in *Ift46*cKO mice. **A** Various postnatal day 18 (P18) WT and collecting duct-conditionally *Ift46* knock-out (Ift46-flox/flox; HoxB7-cre) mouse kidney proteins were analyzed using western blotting. Expression of Limk2 and its target gene, phosphorylated cofilin changed in *Ift46* cKO mouse. p62, as an autophagy marker, and EMT markers also changed in Ift46-deficient mouse. **B** Graphs indicate that relative western blot band intensity ratio after normalization with β-actin as a loading control and phosphorylated cofilin exceptionally normalized with total cofilin. Relative band intensities were measured using ImageJ program. **C** Immunofluorescence images show Limk2 (yellow) and p62 (magenta) expression with cyst-lining epithelial cells in P18-collecting duct-specific Ift46 knock-out mice. Dolichos Biflorus Agglutinin (DBA) (green) labeled renal collecting duct cells. **D** The representative images of immunofluorescence were stained with EMT marker; E-cad labeled epithelial E-cadherin, Vim labeled mesenchymal vimentin. Scale bars in the right bottom are 50 μm. **E** Immunofluorescence staining with LIMK2 (green) and p62 (magenta) in normal and polycystic kidney disease patient tissues. Magnified images of cropped square from original representative images showed below. Scale bars in the right bottom of magnified images are 20 μm. **F** Summary diagram. In regular conditions with Ift46 protein, basal autophagy operates ordinarily and regulates the degradation of some p62 and Limk2 (left side). When Ift46 is absent, the progression of autophagy is disrupted and result in accumulation of p62 and Limk2. Increased Limk2 promotes partial EMT and causes cystogenesis

We firstly checked that Ift46 was diminished in cyst-lining collecting cells of Ift46 conditional knockout mice (Ift46 cKO) within postnatal day 18 (Supplementary Fig. 8). Although E-cadherin showed no difference, not only Limk2 and its target p-Cfl, but also p62 and α-Sma, vimentin as mesenchymal markers was increased in Ift46 cKO (Fig. 6A, B). In a previous study, the reduction of phosphorylated-Ulk1 and LC3B-Ⅱ was reported so early-stage autophagy block by Ift46 absence was demonstrated again [10]. Collecting duct cells consist of enlarged cysts actively expressing Limk2, p62, and Vimentin, these results implied that cyst expansion is closely related with overexpression of Limk2, p62, and partial EMT (Fig. 6C, D). Finally, we observed in human PKD patient kidney tissues, and unsurprisingly high expression with LIMK2 and p62 in cyst-lining collecting duct cells (Fig. 6E). However, cyst lining cells without collecting ducts showed low expression of LIMK2 and p62.

By using open human ADPKD patients’ ATAC-sequencing results, the activity of our target genes was identified (Supplementary Fig. 9A). Within principal cells (PCs) and intercalated cells (ICs) which are comprised collecting duct in PKD, average expression was declined with IFT46, MAP1LC3A (known as LC3A), MAP1LC3B (known as LC3B), and ULK1; moreover, most percentage of expression declined, either. The average expression of SQSTM1 (known as p62) was clearly on the rise in ICs but decrease with percentage and average of expression in PCs. Lastly, the average expression of LIMK2 in PKD was higher than in controls and even the percentage expression in PCs was increased. Furthermore, we examined Limk2 expression in a published microarray dataset (GSE7869) and found that Limk2 expression increased from small to large cysts in human ADPKD (Supplementary Fig. 9B).

Additionally, we attempted to check using ADPKD cyst-lining primary cells, then it was verified that reduced LIMK2 and overexpressed IFT46 decreased the viable ADPKD cell ratio (Supplementary Fig. 10A, B, C). IFT46 knockdown or LIMK2 overexpression promoted α-SMA and vimentin (Supplementary Fig. 10D, E). Finally, LIMK2 and its target p-CFL, and VIM, N-CAD were promoted under IFT46 silencing ADPKD cells (Supplementary Fig. 10F). Subsequently, LIMK2 knockdown extremely decreased the expression of p-CFL, and VIM, N-CAD. The summary of this study is briefly explained in the schematic diagram (Fig. 6F). In normal conditions, Ift46 routinely regulates autophagy, and p62 and Limk2 are also managed at a certain expression in balance. In the Ift46 absence condition, it is hard to organize a complete phagopore for autophagy process, consequently, Limk2 increases. Enhanced Limk2 eventually affects partial EMT promotion and causes renal cyst formation.

## Discussion

Recent studies have suggested that partial EMT is increased in cyst-lining cells in PKD disease [22]. As we expected, our finding data indicated enhanced mesenchymal transition due to Ift46 reduction, however, scant change of epithelial transition. Incidentally, we observed Limk2 increase under Ift46 silencing. Limk2 is proposed as a resolver connecting partial EMT regulatory mechanism of Ift46 because Limk2 is related to the EMT regulation [34]. Consequently, Limk2 and Ift46 dual suppression lulled the promotion of partial EMT phenotypes.

Our prior research revealed that the possibility of autophagy is controlled by Ift46 [10], and association between other IFT complex proteins and autophagy was also discovered [35–38]. We demonstrated that the absence of Ift46 interrupts the autophagy flux, and there is a possibility that Ift46 blocked early autophagy because of p-Ulk1 (S317) decrease and LC3AB-Ⅰ accumulation. Although further observation is required, a series of results show that Ift46 deletion is expected to inhibit autophagy due to abnormalities in the PAS (Pre-autophagosomal Structure) stage that forms vesicles during the autophagy process [39]. Subsequent attempts using autophagy drug treatment revealed that autophagy degrades Limk2. We also found that Limk2 strongly interacts with p62, which was further enhanced under Ift46-deficient conditions. Thus, our results implied that post-translational modification of Limk2 occurs by active autophagy.

Next, we confirmed that Ift46-deficient cells increased tubulogenesis and cystogenesis, which was restored by double silencing of Ift46 and Limk2, additionally, induced autophagy also controlled them. In other words, Ift46 and Limk2 regulate mesenchymal transition and cyst formation dependent of Limk2 and autophagy induction. Our findings for Ift46 and Limk2 by Ift46 conditional deficient mouse and patients with human polycystic kidney disease were verified; the expression tendency of Ift46 and Limk2 was common across all cystic kidney model. In summary, the following proposal is that restoring via Ift46 improvement and Limk2 diminution alleviates kidney cystic disorder.

However, a limitation of this study is that detailed experiments are insufficient for autophagy mechanism related with Ift46 and Limk2 in PKD. Autophagy is an extremely complex process, so it should be possible to reveal the mechanism of autophagy controlled by intraflagellar transport proteins through further research. Nevertheless, the flow is equal to each Limk2 and autophagy affected by the absence of Ift46. Although the biological role of autophagy in PKD pathogenesis remains controversial, numerous studies have shown that autophagy is dysregulated in PKD. Thus, identifying pathological target proteins that are specifically regulated by autophagy is critical for understanding PKD pathogenesis. In this context, we identify LIMK2 as a pathological target of autophagy in PKD.

In conclusion, this research provides evidence for the function of Ift46 to inhibit Limk2 via autophagy control including EMT progression and renal cystogenesis. Recent research reported that the possibility non-ciliary function of IFT proteins in PKD but accurate mechanisms are still lacking [40]. Since it is the first approach that Ift46 presents a cilia-independent role, it will be of great help to in-depth understanding of the mechanism of PKD.

## Conclusions

Although it is widely known that loss of Ift46 induces renal cyst formation, the extraciliary role of Ift46 has been little discussed. Here we propose that Ift46 is required for basal autophagy in renal collecting duct cells, specifically regulates autophagic adaptor, p62. Furthermore, for the first time, our results confirmed that Ift46 defect increases translated Limk2. Various autophagy drugs affect the stability of Limk2, and an interaction between Limk2 and p62 is also identified. These findings therefore imply that Ift46 deficiency increases Limk2 through aberrant autophagy. Disruption of the IFT46–autophagy–LIMK2 axis induces partial EMT and cyst formation, supporting a role for LIMK2 in PKD-associated cystogenesis.

## Supplementary Information

Supplementary Material 1.

Supplementary Material 2.

## Data Availability

The datasets used and/or analysed during the current study are available from the corresponding author on reasonable request.

## Abbreviation

Ift46: Intraflagellar transport protein 46
Limk2: LIM domain kinase 2
EMT: epithelial–mesenchymal transition
PKD: polycystic kidney disease
