# Supplementary material for: Ift46 deficiency causes renal cyst via enhanced Limk2 through lack of autophagy flux
Source: Cell Commun Signal. 2026 Feb 12;24:185. doi: 10.1186/s12964-026-02715-4 (PMC13001273; doi:10.1186/s12964-026-02715-4)
Supplement: Supplementary file 1 — Supplementary Material 1. [file 12964_2026_2715_MOESM1_ESM.pdf]

Supplemental Data

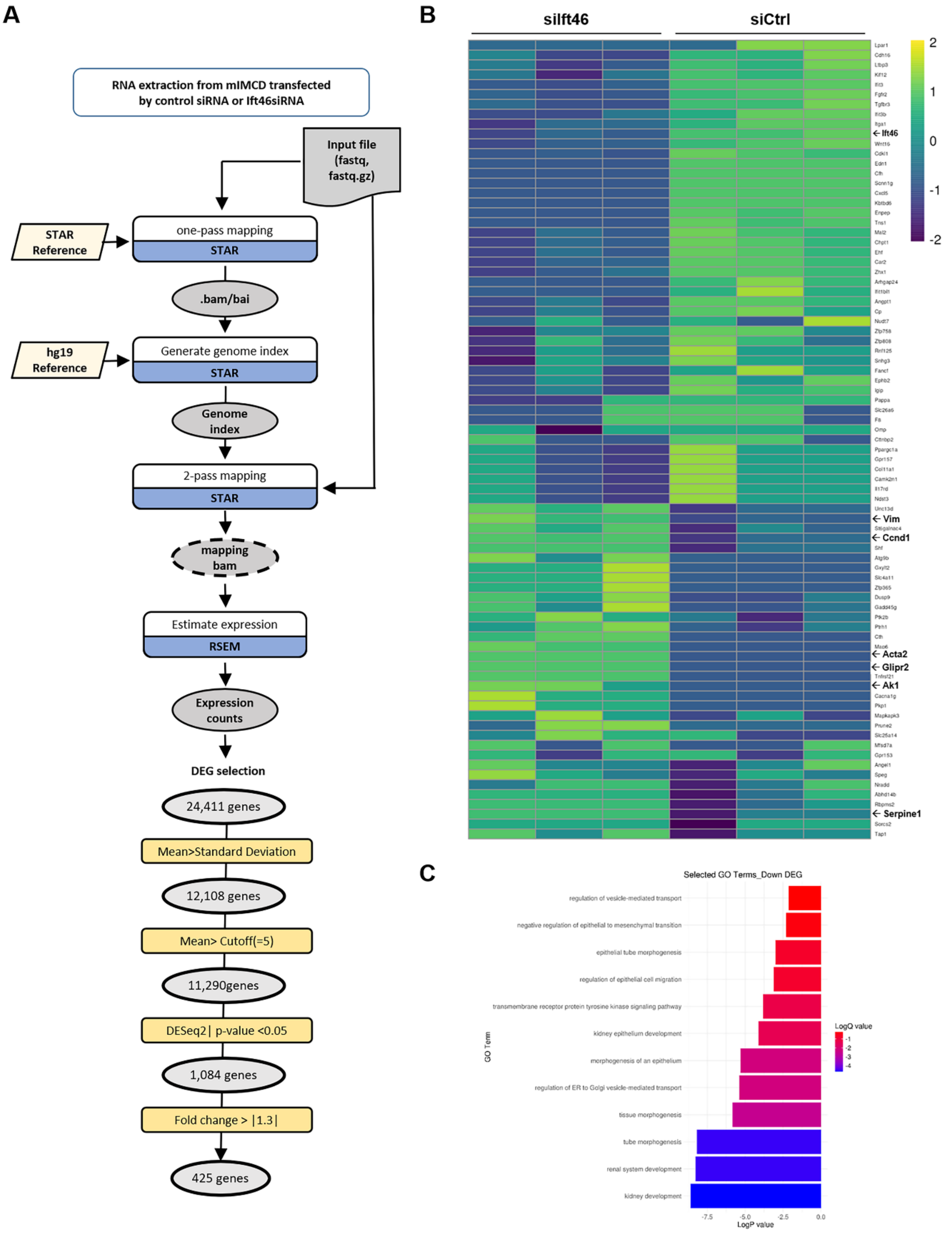

Supplementary Figure 1. Systemic analysis of total RNA sequencing using Ift46-silenced IMCD cells. (a) Pipeline of total RNA-sequencing. (b) Heatmap was drawn using the pheatmap (v1.0.12) of the R package with the genes selected as 1.7% upregulated and downregulated DEGs. Genes with

mesenchymal significance were indicated in bold and with arrows. (c) Graph suggests GO term analysis with down-regulated DEG extracted from Ift46 KD IMCD. Length of bar graph means log P value and colors indicate the value of log Q.

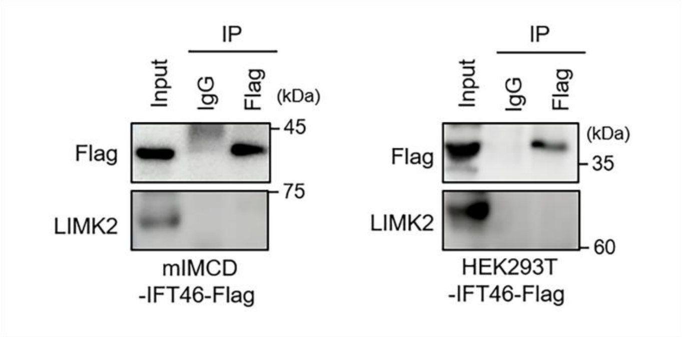

Supplementary Figure 2. LIMK2 does not interact with p62. Co-IP assay was performed with flag-tagged IFT46 transfected mIMCD (left) HEK293T cells (right).

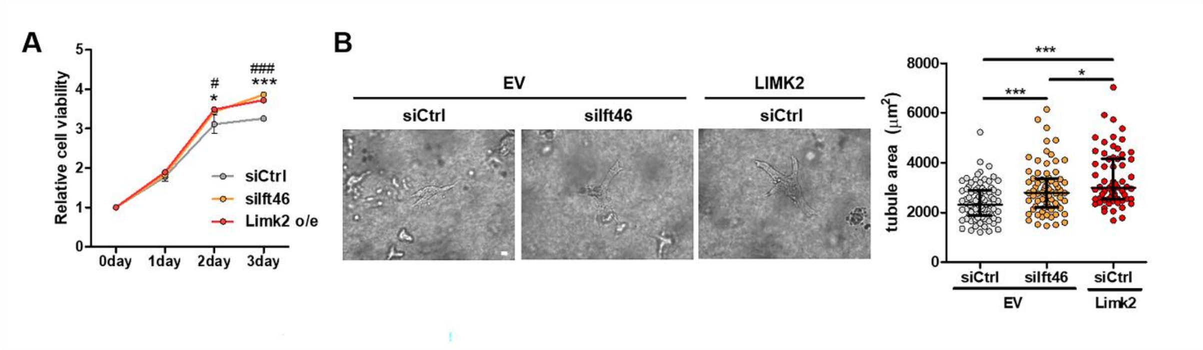

Supplementary Figure 3. LIMK2 overexpression induces phenotypes similar to those observed upon IFT46 loss. (**a**) Cell viability and (b) *in vitro* tubule formation assay in Ift46 siRNA transfected and Limk2 overexpressing mIMCD cells.

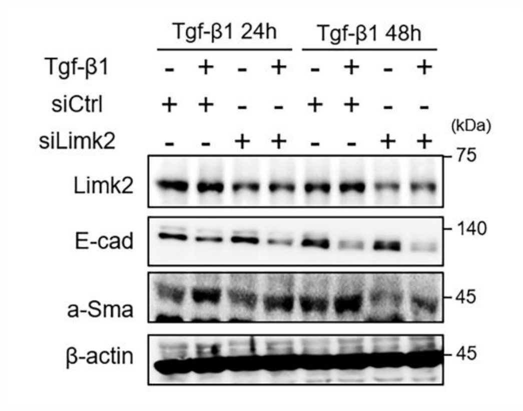

Supplementary Figure 4. Limk2 regulates partial epithelial-to-mesenchymal transition. α-Sma changes under silenced Limk2 with TGF-β1 for 24 or 48 h using western blotting analysis.

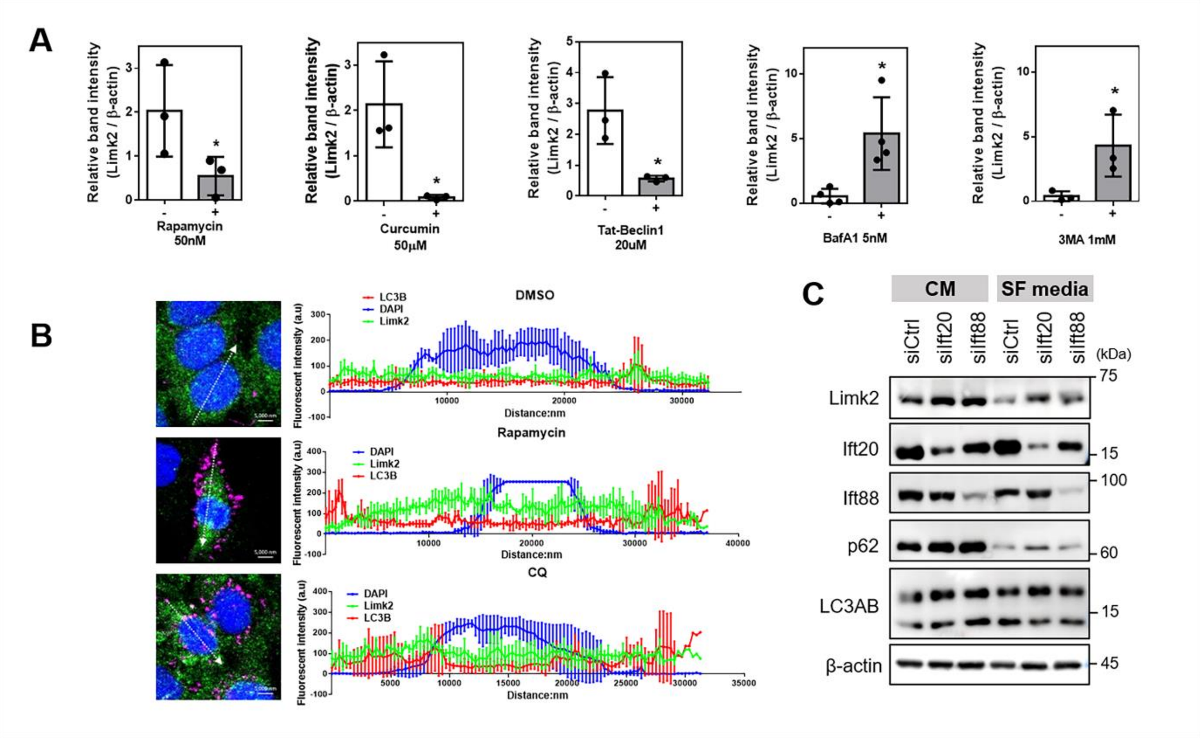

Supplementary Figure 5. Ift46 and Limk2 were closely related to autophagy. (a) Graphs showed that relative band intensity of Limk2 under treating autophagy regulating drugs. (b) The right graphs showed the fluorescent intensity observed along the white dotted arrow of the left immunofluorescence images. Measurement values of three cells of similar size were plotted, and X-axis is distance (nm) and Y-axis is relative fluorescent intensity. (c) Immunoblotting analysis indicated that increased Limk2 expression under IFT20 KD and IFT88 KD with or without FBS. *P < 0.05, **P < 0.01

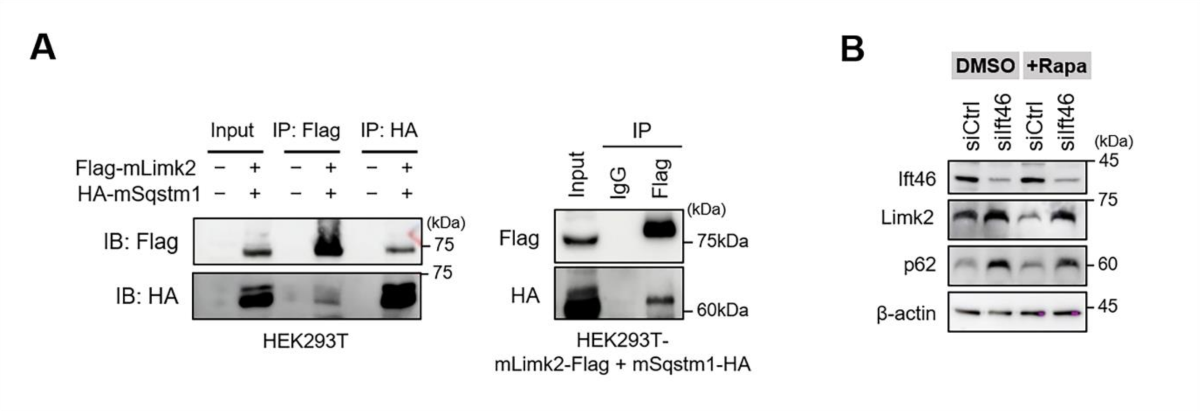

Supplementary Figure 6. Relationship between Limk2 and p62. (a) Co-IP assay is performed with flag-tagged mouse Limk2 and HA-tagged mouse p62/Sqstm1 transfected HEK293T cells. (b) Protein expressions of Limk2 and p62 were affected by Ift46 knocked down with rapamycin-induced autophagy. Rapamycin was treated with 50 nM for 4 hours.

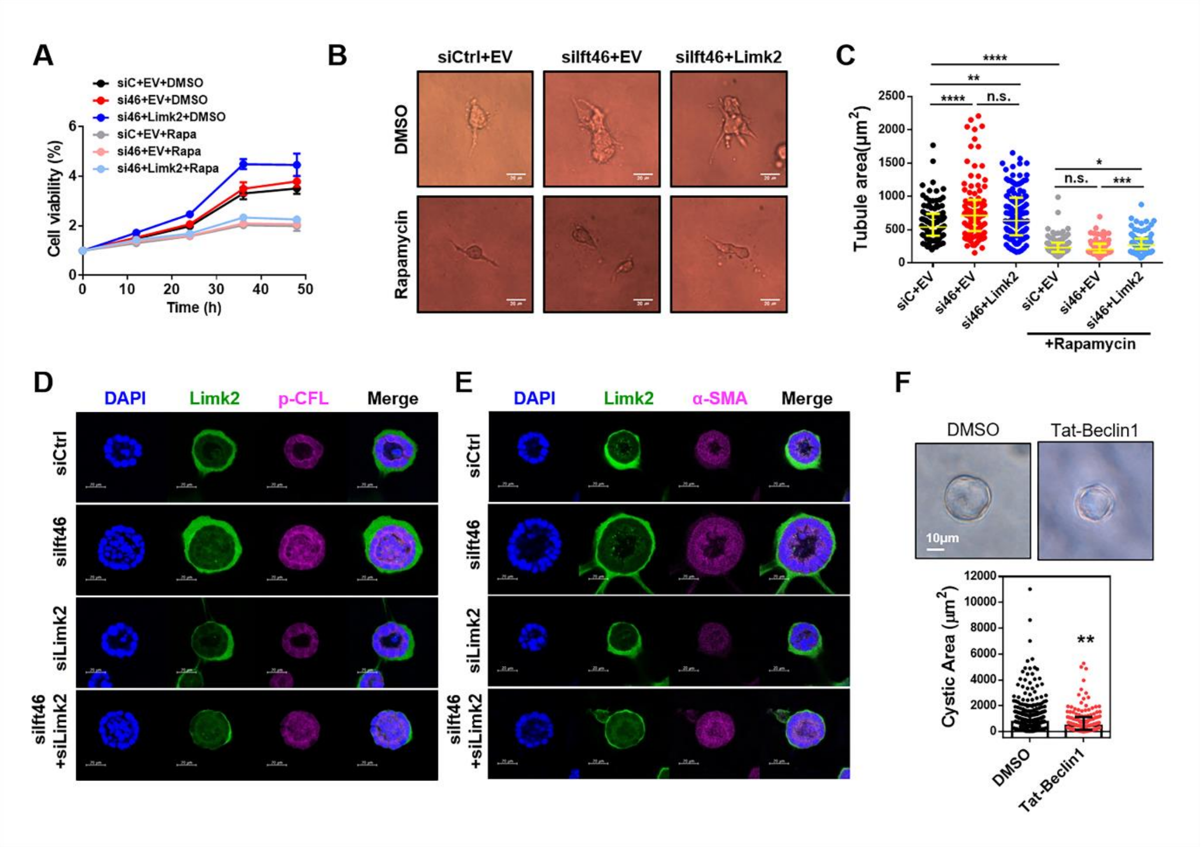

Supplementary Figure 7. Increase of mesenchymal transition characteristics and cystogenesis by over-expressed Limk2 under altering autophagy flux. (a–c) Data show increased EMT phenotypes through cell viability assay using CCK-8, and tube morphogenesis under induced autophagy with Ift46 silencing and Limk2 overexpression in mIMCD cells. Rapamycin treatment was 50 nM for 4 h. (a) Quantified viable cell ratio under identical conditions for 12 h. (b) Representative images of tubular morphogenesis under identical conditions. (c) Tubule size was quantified and is shown in the graph. (d-e) Photographs of representative ICC analysis of Limk2 and a-Sma or p-CFL in mIMCD cells. These images show representative results of double-immunostaining of Limk2 (green) and a-Sma or p-Cfl (red) in mIMCD cells. Scale bars were in the bottom of images. (f) Representative images showing differences in cystic area size after treatment with 20 μM Tat-Beclin1 for 4 hours. Scale bars in the lower left corners represent 10 μm. The graph below quantifies cystic area changes following Tat-Beclin1-induced autophagy. **P < 0.01, ***P < 0.001. n.s. means no-significant.

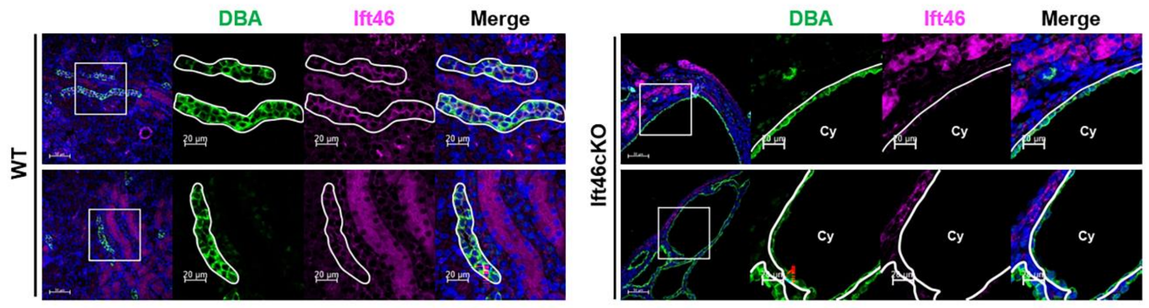

Supplementary Figure 8. Verification of collecting-duct-specific Ift46 deficient conditional mouse. Immunofluorescence images displayed the expression of Ift46 nearby collecting duct, labeled DBA-red. The white lines indicated Ift46 expression with collecting duct cells. ‘Cy’ means renal cyst derived from collecting duct. Scale bars in leftmost images were 50 μm and magnified images were 20 μm.

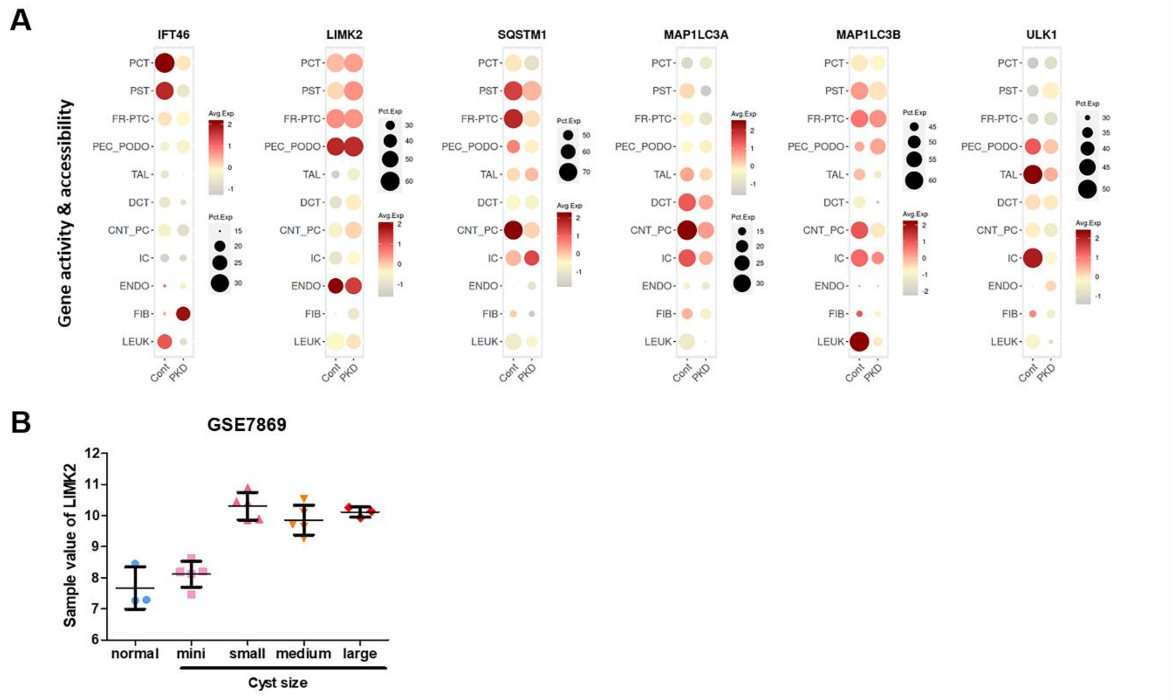

Supplementary Figure 9. Expression verification of Ift46-autophagy-Limk2 axis using open published human ADPKD scATAC-sequencing data. (a) Gene activity and accessibility of IFT46, LIMK2, p62/SQSTM1, and another autophagy marker MAP1LC3A, MAP1LC3B and ULK1. Gene activity was compared by focusing on ICs (intercalated cells) belonging to collecting ducts among various tubules. (b) LIMK2 expression analysis in GSE7869.

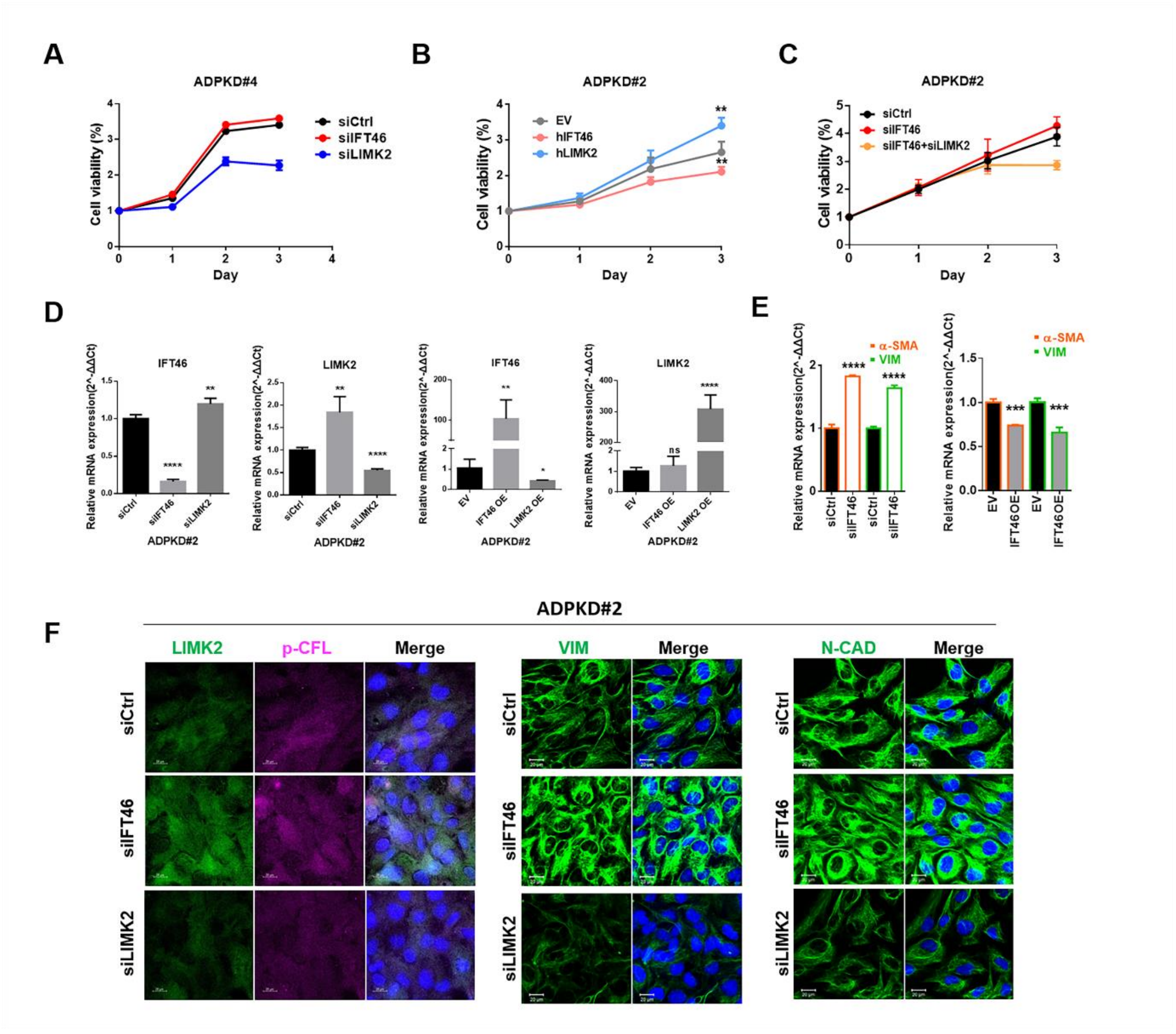

Supplementary Figure 10. Effect of Ift46 and Limk2 on ADPKD patient cyst-lining cells. (a-c) Quantified viable cell ratio under identical condition in terms of 24 hours. IFT46 knock-down and overexpressed LIMK2 increase cellular viability whereas LIMK2 knock-down and overexpressed IFT46 decrease. (d) Relative mRNA expression graphs by qRT-PCR confirmed IFT46 and LIMK2 expression following knockdown or overexpression experiments. (e) Quantitative real-time PCR validation of mesenchymal marker genes, α-sma and vimentin, under alteration of IFT46 expression. (f) Immunofluorescence staining images represent changes of LIMK2, phosphorylated CFL, VIMENTIN, and N-CADHERIN under IFT46 or LIMK2 silencing in ADPKD cells. DAPI stained blue. Scale bars in the left bottom are 20 μm.

1. siRNA

Product	Company	Catalog#
IFT46 siRNA (m)	Santa Cruz Biotechnology	sc-108246
LIMK-2 siRNA (m)	Santa Cruz Biotechnology	sc-35813
SQSTM1/p62 siRNA (m)	Santa Cruz Biotechnology	sc-29828
IFT20 siRNA (m)	Santa Cruz Biotechnology	sc-146174
IFT88 siRNA (m)	Santa Cruz Biotechnology	sc-146178

2. Autophagy Drug

Product	Company	Catalog#	Treatment
Rapamycin	Sigma-Aldrich	R8781	50 nM for 4 h
Curcumin	Sigma-Aldrich	C1386	5–50 µM for 4 h
Earle's balanced salt solution (EBSS)	WelGENE	LB 002-03	2 h
3-methyladenine	Sigma-Aldrich	189490	0.5–2 mM for 4 h
chroloquine (CQ)	Sigma-Aldrich	C6628	30–50 µM for 4 h
bafilomycin A1	Sigma-Aldrich	B1793	1–5 nM for 4 h
Tat-Beclin-1	Anaspec	AS-65467	0.5-2 µM for 24 h

3. Antibody

Product	Company	Catalog#	Application
Anti-IFT46 antibody	Abcam	ab122422	WB, ICC
Anti-LIMK-2 (A-12)	Santa Cruz Biotechnology	sc-365414	WB, ICC
SQSTM1/p62 Antibody	Cell Signaling Technology	5114S	WB, ICC
SQSTM1/p62 antibody (D-3)	Santa Cruz Biotechnology	sc-28359	WB, ICC
Anti-α-Smooth Muscle Actin (D4K9N) XP® Rabbit mAb	Cell Signaling Technology	19245	WB, ICC
Vimentin (D21H3) XP® Rabbit mAb	Cell Signaling Technology	5741	WB, ICC

N-Cadherin (13A9) Mouse mAb	Cell Signaling Technology	14215	WB
Purified Mouse Anti-E-Cadherin	Cell Signaling Technology	610181	WB, ICC
Rabbit anti-Cytoskeletal Actin Antibody	BETHYL	A300-491A	WB, ICC
LC3A/B (D3U4C) XP® Rabbit mAb	Cell Signaling Technology	12741	WB, ICC
ULK1 (D8H5) Rabbit mAb	Cell Signaling Technology	8054	WB
Phospho-ULK1 (Ser317) Rabbit mAb	Cell Signaling Technology	37762	WB
Goat anti-rabbit IgG	Enzo	ADI-SAB-300-J	WB
Goat anti-mouse IgG	Enzo	ADI-SAB-100-J	WB
Cofilin (D3F9) XP® Rabbit mAb	Cell Signaling Technology	5175	WB
Phospho-Cofilin (Ser3) (77G2) Rabbit mAb	Cell Signaling Technology	3313	WB, ICC
CEP164 Polyclonal antibody	Proteintech	22227-1-AP	ICC
IFT20 Polyclonal antibody	Proteintech	13615-1-AP	WB
IFT88 Polyclonal antibody	Proteintech	13967-1-AP	WB
Normal Mouse IgG	Merck Millipore	12-371	IP
Monoclonal Anti-FLAG® M2	Sigma-Aldrich	F1804	IP
Anti-FLAG	Sigma-Aldrich	F7425	WB
Anti-HA.11 clone 16B12	Biolegend	901515	IP
HA-Tag (C29F4)	Cell Signaling Technology	3724S	WB
Goat anti-Mouse IgG (H+L), Alexa Fluor™ 488	Invitrogen	A-11029	ICC

Goat anti-Mouse IgG (H+L), Alexa Fluor™ 594	Invitrogen	A-11032	ICC
Goat anti-Rabbit IgG (H+L), Alexa Fluor™ 488	Invitrogen	A-11034	ICC
Goat anti-Rabbit IgG (H+L), Alexa Fluor™ 546	Invitrogen	A-11035	ICC
Goat anti-Rabbit IgG (H+L), Alexa Fluor™ 594	Invitrogen	A-11037	ICC
DAPI	Sigma-Aldrich	D9542	ICC

Supplementary Table 1. siRNAs, autophagy drugs, and antibodies used in this study.
