# Supplementary figures and images for: Ift46 deficiency causes renal cyst via enhanced Limk2 through lack of autophagy flux
Source: Cell Commun Signal. 2026 Feb 12;24:185. doi: 10.1186/s12964-026-02715-4 (PMC13001273; doi:10.1186/s12964-026-02715-4)

Figure 1

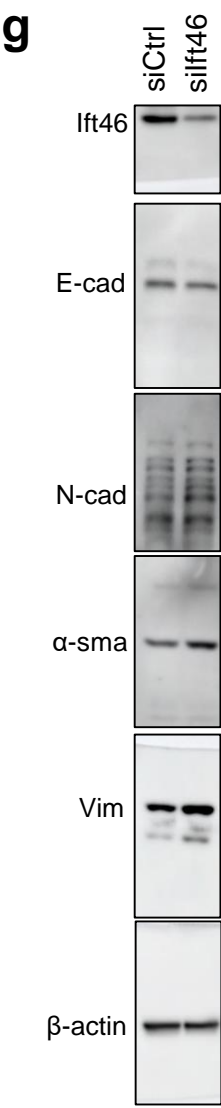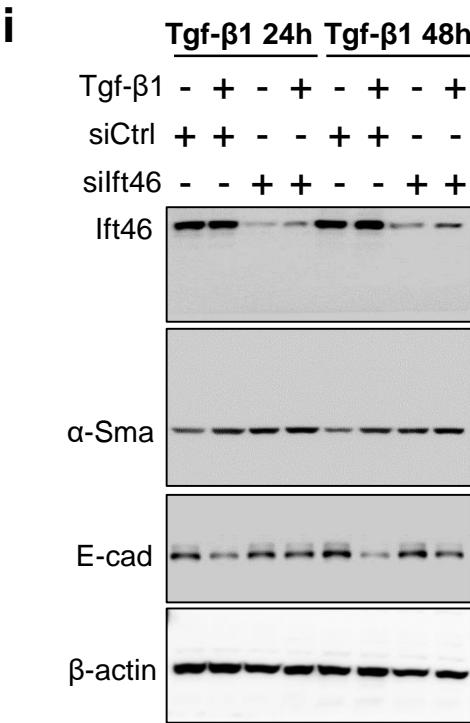

Figure 2

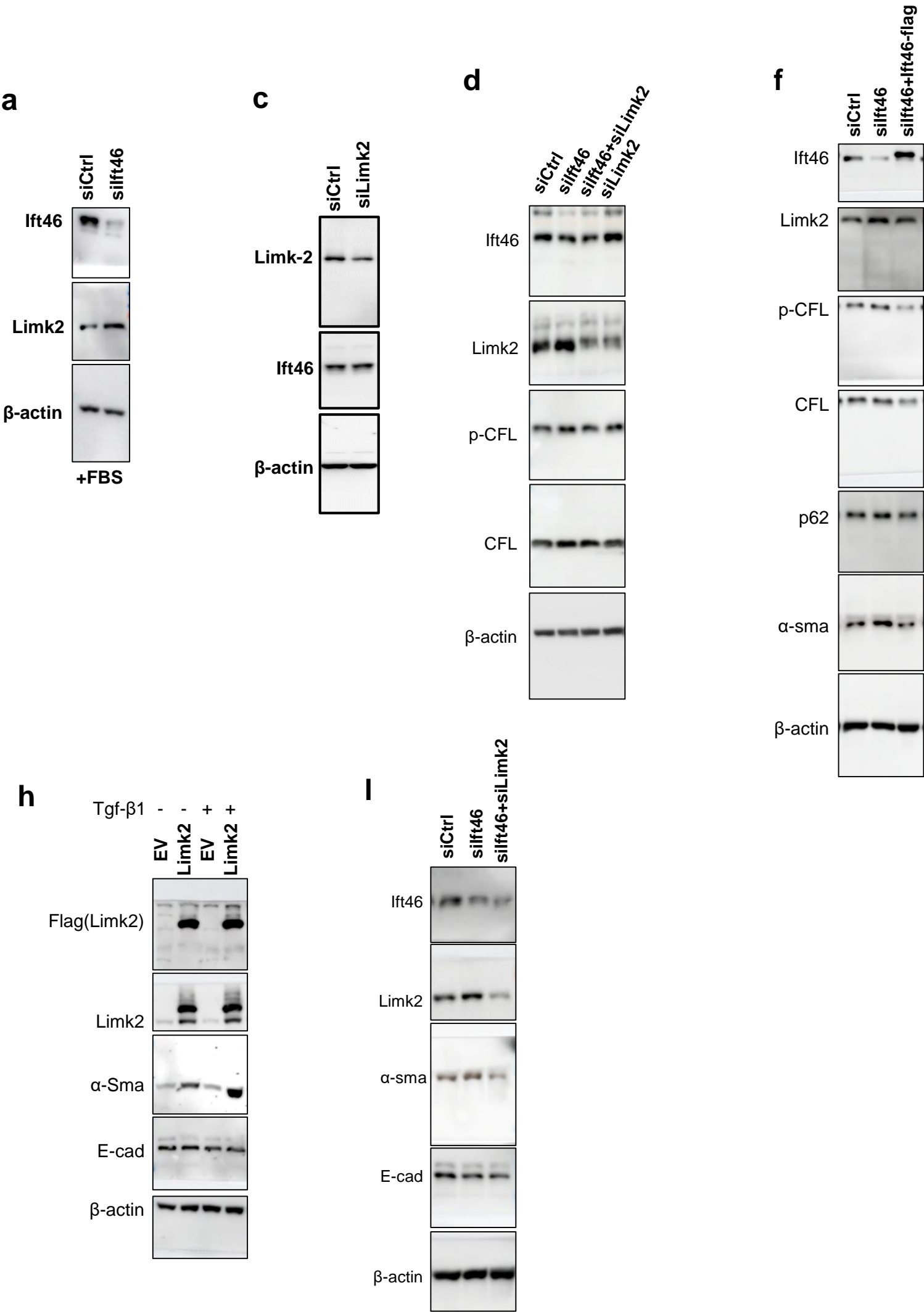

Figure 3

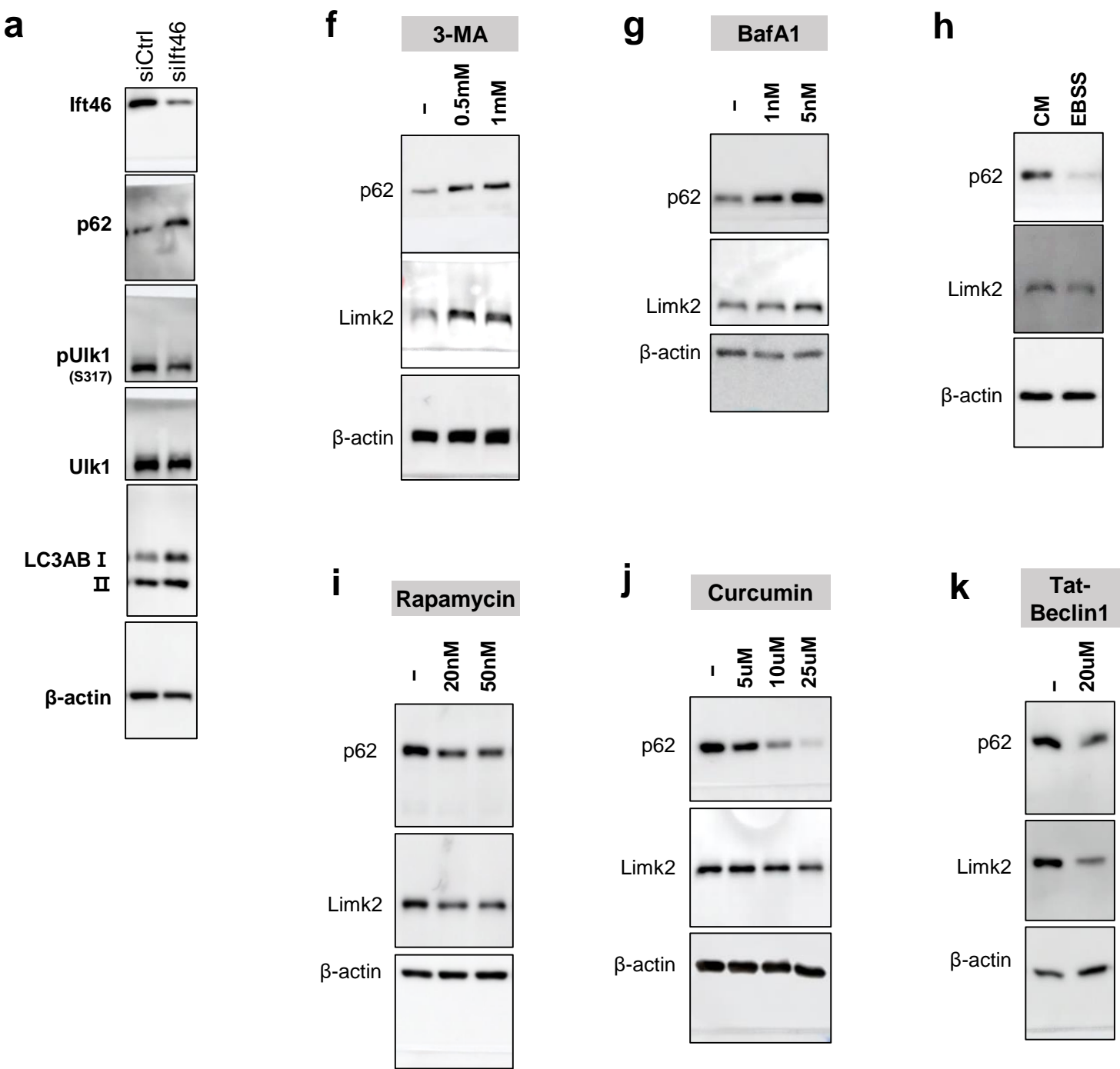

Figure 4

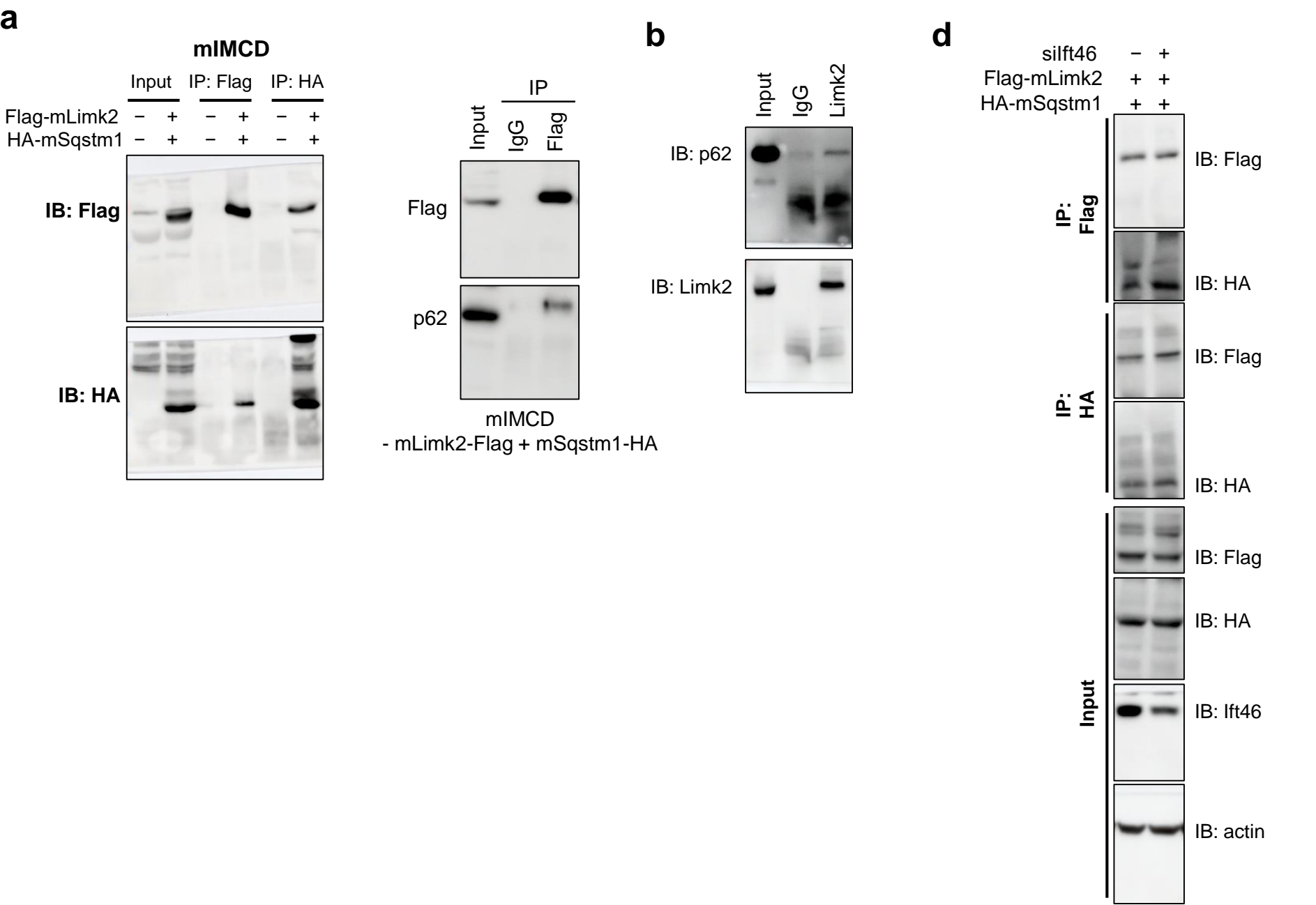

Figure 5

a

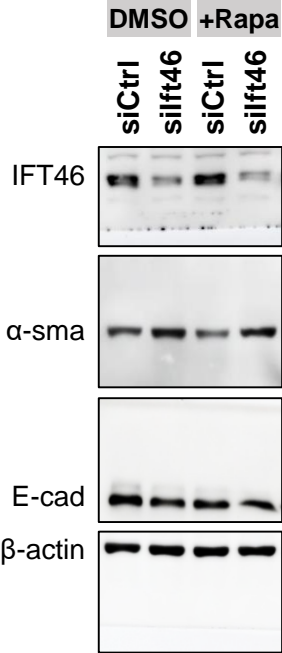

j

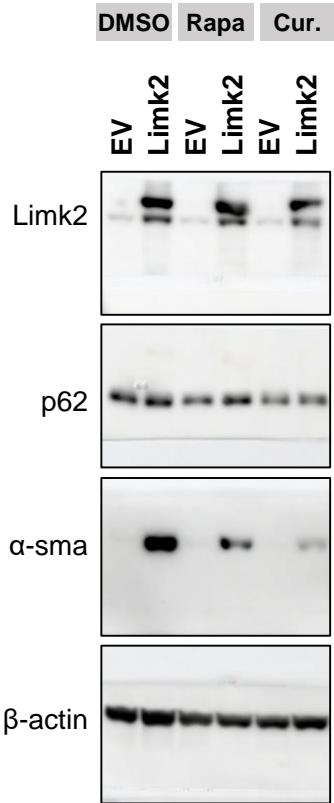

Figure 6

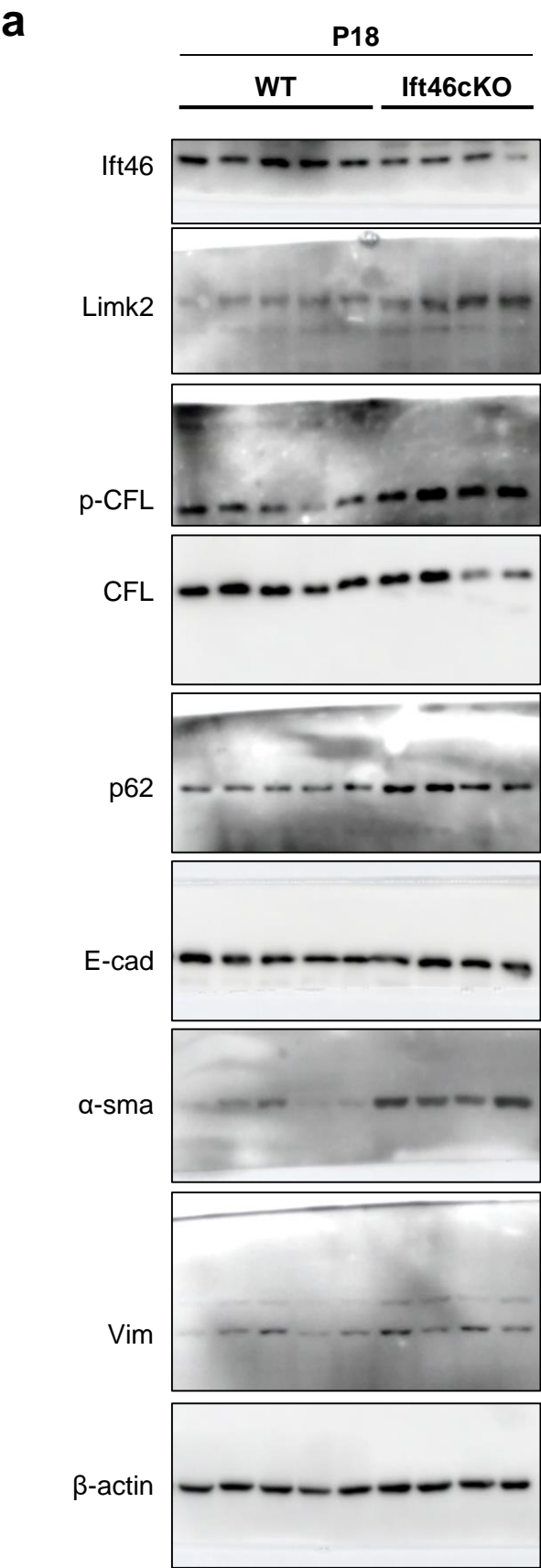

## Sup Fig 2

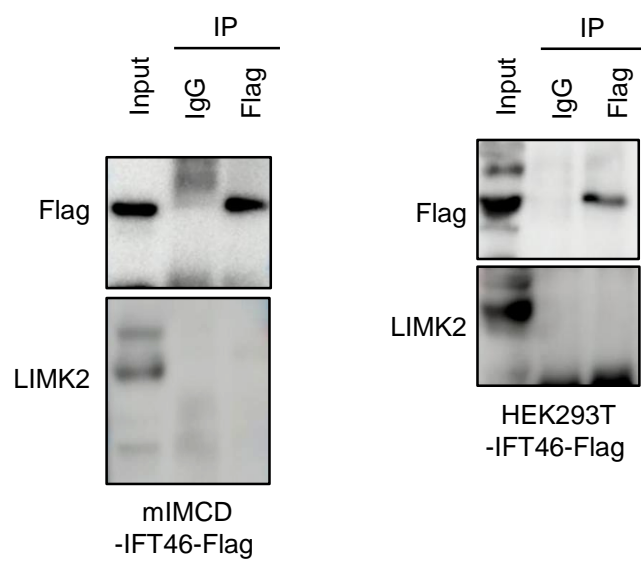

## Sup Fig 4

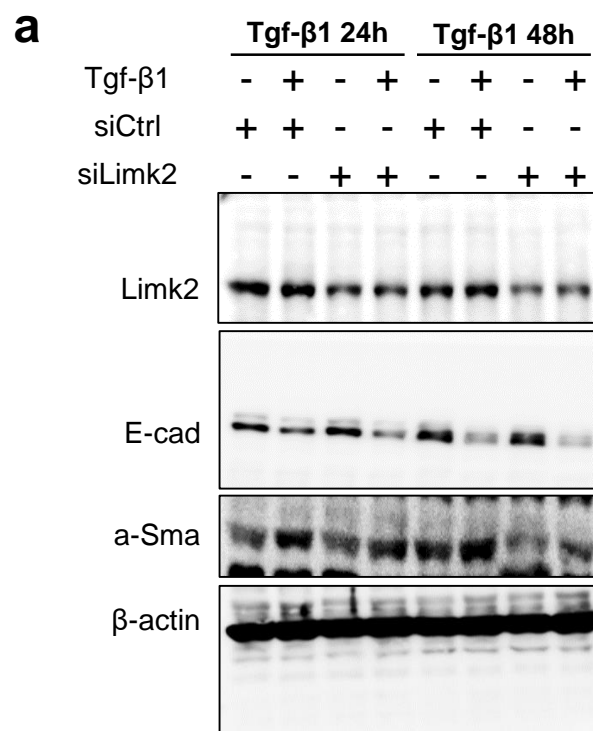

Sup Fig 5

C

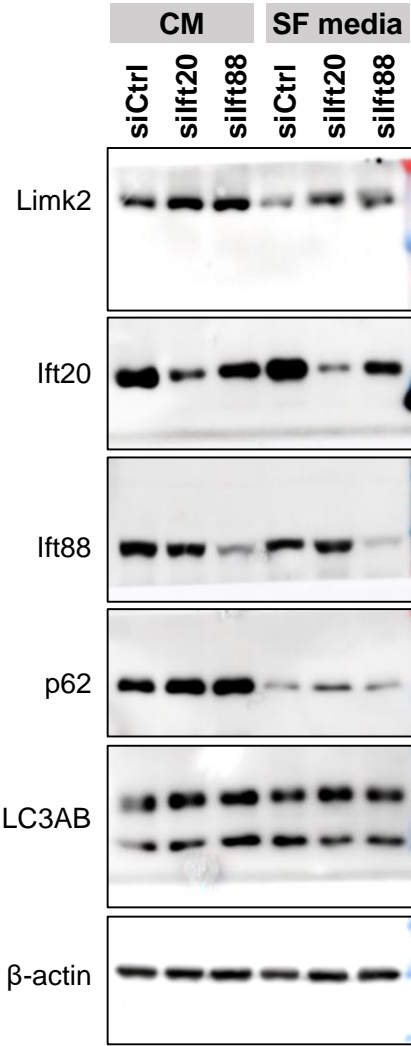

Sup Fig 6

a

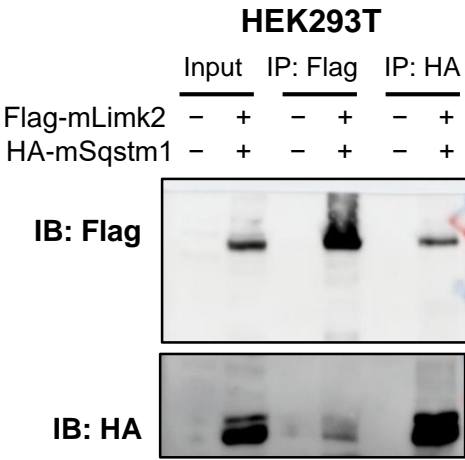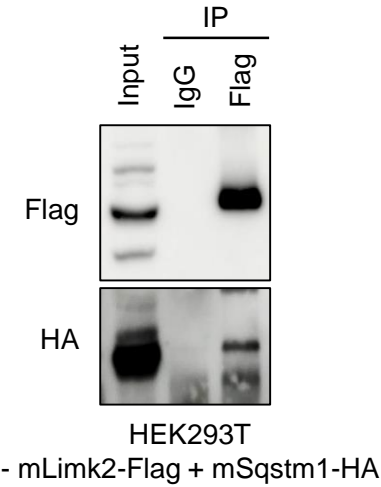

b

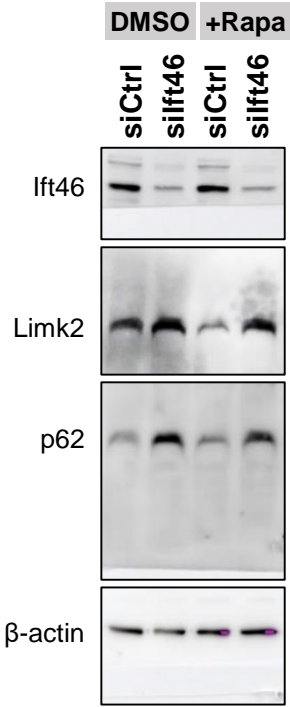

Supplement: Supplementary file 2 — Supplementary Material 2. [file 12964_2026_2715_MOESM2_ESM.pdf]
